# Design, Synthesis, and Antileukemic Evaluation of a Novel Mikanolide Derivative Through the Ras/Raf/MEK/ERK Pathway

**DOI:** 10.3389/fphar.2022.809551

**Published:** 2022-05-20

**Authors:** Qing Rao, Kaiqiang Xie, Krishnapriya M. Varier, Lei Huang, Jingrui Song, Jue Yang, Jianfei Qiu, Yubing Huang, Yan Li, Babu Gajendran, Yanmei Li, Sheng Liu

**Affiliations:** ^1^ State Key Laboratory for Functions and Applications of Medicinal Plants, Guizhou Medical University, Guiyang, China; ^2^ The Key Laboratory of Chemistry for Natural Products of Guizhou Province and Chinese Academic of Sciences, Guiyang, China; ^3^ School of Pharmaceutical Sciences, Guizhou Medical University, Guiyang, China

**Keywords:** mikanolide derivatives, 3G, sesquiterpene, leukemia, apoptosis, cell cycle, K562

## Abstract

Chronic myeloid leukemia (CML) accounts for a major cause of death in adult leukemia patients due to mutations or other reasons for dysfunction in the ABL proto-oncogene. The ubiquitous BCR–ABL expression stimulates CML by activating CDK1 and cyclin B1, promoting pro-apoptotic, and inhibiting antiapoptotic marker expression along with regulations in RAS pathway activation. Thus, inhibitors of cyclins and the RAS pathway by ERK are of great interest in antileukemic treatments. Mikanolide is a sesquiterpene dilactone isolated from several Asteraceae family *Mikania* sp. plants. Sesquiterpene dilactone is a traditional medicine for treating ailments, such as flu, cardiovascular diseases, bacterial infections, and other blood disorders. It is used as a cytotoxic agent as well. The need of the hour is potent chemotherapeutic agents with cytotoxic effects inhibition of proliferation and activation of apoptotic machinery. Recently, ERK inhibitors are used in clinics as anticancer agents. Thus, in this study, we synthesized 22-mikanolide derivatives that elucidated to be potent antileukemic agents *in vitro*. However, a bioactive mikanolide derivative, 3g, was found with potent antileukemic activity, through the Ras/Raf/MEK/ERK pathway. It can arrest the cell cycle by inhibiting phosphorylation of CDC25C, triggering apoptosis, and promoting DNA and mitochondrial damage, thus suggesting it as a potential chemotherapeutic agent for leukemia patients.

## Introduction

Chronic myeloid leukemia (CML) is a myeloproliferative syndrome of primitive hematopoietic progenitor cells. It accounts for around 20% of leukemic cases reported in adults. The major triggers for this type of leukemia are the BCR–ABL tyrosine kinase dysfunctions due to mutations or other causes (Ben-Neriah et al., 1986; Jackson and Baltimore, 1989; Branford et al., 2002). The ABL is a proto-oncogene that coordinates many cellular activities, such as cell differentiation, adhesion, DNA damage response, and apoptosis. This BCR–ABL gene, when expressed, monitors the expression of cyclin-dependent kinase 1 (CDK1). Therefore, the mutations in this gene cause DNA damage and the activation of RAS pathways. BCR–ABL expression promotes CML due to damaged cell adhesion capacity cells (Overduin et al., 1992; Musacchio et al., 1994; Willis et al., 2005; Vaidya et al., 2015), causing uncontrolled blood cells’ growth in CML patients. Thus, recently the chemotherapeutic drugs such as imatinib and several tyrosine inhibitors (Wan et al., 2016; Liu et al., 2020; Maiti et al., 2020) focusing on BCR–ABL gene regulation and related pathways are of great attraction for leukemia treatment researchers.

Deregulation of the ERK pathway usually happens due to genetic adjustments of several crucial molecules of this pathway. In contrast, uncontrolled leukemic proliferation can occur due to diminished compassion to apoptosis-initiating factors or due to chemo-resistance correlated to pro-survival molecules activation. Nevertheless, inactivated ERK pathway molecules could considerably alter reactions toward small molecule inhibitors. Moreover, the ERK pathway is inhibited by many usual chemotherapeutic drugs used for leukemia (McCubrey et al., 2007; McCubrey et al., 2008a; Steelman et al., 2008). Mikanolide is a sesquiterpene dilactone isolated from many kinds of Mikania plants. It has been used as a folk medicine for a long time and has been paid attention by researchers due to its antibacterial, antitumor, antimicrobial, and cytotoxic functions (Bohlmann et al., 1984; Gutierrez et al., 1985; Pickman, 1986; Ysrael and Croft, 1990; Facey et al., 1999; Ahmed et al., 2001; Zhuang et al., 2010). Sesquiterpenes are biosynthesized in the plant endoplasmic reticulum by farnesyl pyrophosphate as colorless lipophilic compounds. Sesquiterpenes are a 15-carbon backbone molecule with diverse structural orientations and some functional cyclic ones. The most bioactive compounds having cardiovascular effects inhibit iNOS and NF-κB (Giordano et al., 1990). Another study revealed parthenolide— a germacranolide sesquiterpene lactone—inhibits JNK activation in CNE1, COLO205, HELA, HBL-100, and MDA-MB-231 cells. Parthenolide sensitizes TNF-related apoptosis-inducing ligand (TRAIL) proteins offering anticancer action (Nakshatri et al., 2004; Zhang et al., 2004; Guzman et al., 2005). Consequently, as treatment regimens for CML, novel mikanolide derivatives that can act as inhibitors of abnormal cell growth with reduced side effects are investigated. Likewise, the ERK pathway inhibitors can control the sensitivity as well as resistance in leukemia treatment. Thus, in this study, we synthesized 22-mikanolide derivatives that can be antileukemic agents by targeting inhibition of cell proliferation signaling molecules, thus suggesting them as a potential chemotherapeutic agent for leukemia patients.

## Materials and Methods

### Synthesis of Compounds

Proton nuclear magnetic resonance (^1^H NMR) spectra were recorded using Bruker AV 400 MHz or 700 MHz spectrometers. Proton chemical shifts are reported in parts per million (d scale) and are referenced using residual protium in the NMR solvent [CDCl_3_: δ 7.26 (CHCl_3_), DMSO-*d*6: d 2.54 (DMSO)]. Data are reported as follows: chemical shift [multiplicity (s = singlet, d = doublet, dd = doublet of doublets, ddd = doublet of doublet of doublets, t = triplet, q = quartet, m = multiplet, and br s = broad singlet), coupling constant(s) (Hz), and integration]. Carbon-13 nuclear magnetic resonance (^13^C NMR) spectra were recorded using Bruker AV 100 MHz or 151 MHz or 176 MHz spectrometers. Carbon chemical shifts are reported in parts per million (d scale), and are referenced using the carbon resonances of the solvent [CDCl_3_: δ 77.0 (CHCl_3_), DMSO-*d*6: δ 40.45 (DMSO)]. Data are reported as follows: chemical shift [multiplicity (if not singlet) and assignment (Cq = fully substituted carbon)]. High resolution mass spectra (HRMS) were documented on a Waters SYNAPT G2 using an electrospray (ESI) ionization source. Column chromatography was performed on silica gel (400–500 mesh) eluting with ethyl acetate and petroleum ether. TLC was performed on glass-backed silica plates, and visualized by UV light and I_2_ products.

A mixture of mikanolide or deoxymikanolide (1.0 mmol) and an appropriate aromatic iodide (1.1 mmol) was refluxed at 115°C using palladium (II) ferrocene (0.01 mmol) and DIPEA (3.0 mmol) in dry toluene (1 ml) under air for 6–12 h. A TLC monitor was used until the reaction was complete, then the reaction mixture was allowed to cool to room temperature, water (10 ml) was added, and the resultant mixture was extracted with ethyl acetate (15 ml × 3). The separated organics were dried over Na_2_SO_4_. Later it was filtered. The filtrate was concentrated under reduced pressure. The obtained crude residue was purified by silica flash chromatography (500:1 to 100:1, dichloromethane/methanol) to afford the corresponding aryl-substituted parthenolide as a solid (65–195 mg) in 15–45% isolated yield.

### Reagents

Roswell Park Memorial Institute 1640 (RPMI-1640, Gibco) was purchased from Thermo Fisher Scientific (Shanghai, China). Fetal bovine serum (FBS) was purchased from the VACCA Biologics LLC. (United States). Dimethyl sulfoxide (D8371), bovine serum albumin (A8020), BCA protein assay kit (PC0020), thiazolyl blue tetrazolium bromide (M8180), and color mixed protein marker (PR 1920) were obtained from Solarbio Life Sciences (Beijing, China). Annexin V-FITC apoptosis detection kit was purchased from BD (United States, Cat. No. 556547). JC-1 mitochondrial membrane potential detection kit (C2006), Hoechst staining kit (C0003), reactive oxygen species assay kit (S0033), SDS-PAGE gel preparation kit (P0012AC), cell lysis buffer (P0013), SDS-PAGE sample loading buffer, 5X (P0015L), and transfer buffer (P0021B) were purchased from Beyotime Biotechnology (Shanghai, China). Ras (ab52939), Bim (ab32158), ERK (ab184699), p-ERK (ab32538), p-PKCδ (ab76181), PKCδ (ab182126), cyclin B1 (ab32053), CDK1 (ab133327), p90RSK(ab32413), caspase 8 (ab25901), Bcl-2 (ab32124), Bcl-xl (ab2568), and p-γH2AX (ab11174) were purchased from Abcam (Abcam, Cambridge, United Kingdom); BID (#2002), P-B-Raf (#2696), P-MEK1/2 (#9154), MEK1/2 (#8727), PARP (#9542), P-CDC25C (#4901), CDC25C (#4688), p21 (#2947), Bad (#9292), Phospho-CDC2 (#4539), caspase 3 (#9662), cleaved caspase 3 (#9661), cleaved caspase 9 (#7237), and c-FLIP (#56343S) were purchased from Cell Signaling Technology; p27 (380960), caspase 9 (381336), and GAPDH (301341) were purchased from Zen Bioscience, China; B-Raf (AF6171) was purchased from Affinity Biosciences, United States .

### Cell Culture

HEL (erythroleukemia), K562 (chronic myeloid leukemia), CEM-C7H2 (T-cell acute lymphoblastic leukemia), and HL7702 (normal hepatic cell line) were purchased from ATCC, Manassas, VA, United States . The cells were cultured in RPMI 1640 medium supplemented with 5% FBS at 37°C in a CO_2_ incubator (5% CO_2_ and 95% air, 95% humidity), as per standard conditions of passage.

### Cell Viability Assays

The cell viability assay was performed by treating different concentrations of compounds on HEL, K562, and CEM-C7H2 cells to find their IC_50_ values. The control groups were treated with 0.1% DMSO or compounds. After 72 h treatment, the cells were added with an MTT solution of 20 µl (5 mg/mL) for 4 h. Later, the 96 well-plates were centrifuged at 2,500 rpm for 20 min and discarded the medium, then DMSO (160 µl) was used to dissolve formazan crystals. The resulting solution was determined using absorbance at 490 nm (BioTek, Winooski, VT, United States). The cell morphology was analyzed under an inverted microscope (Nikon) and photographed (Gajendran et al., 2020a).

### Flow Cytometric Analysis

Cells were seeded at 3 × 10^6^ cells per 60 mm dish in 3 ml medium with different concentrations of 3g (0.3, 0.5, and 1 µM). After growing for 24 and 48 h, the cells were harvested, washed twice with cold PBS, and then the cells were resuspended in 1X binding buffer at a concentration of 1 × 10^6^ cells/ml. Later, 100 µl of cells (1 × 10^5^) were transferred to a 2 ml Eppendorf tube, added with 5 µL of FITC Annexin V and PI (BD FITC Annexin V apoptosis detection kit I) in each tube. After gently vortexing the cells and incubating for 15 min at RT (25°C) in the dark, 400 µl of 1X binding buffer to each tube was added and analyzed by flow cytometry within 1 h (ACEA Biosciences Inc. San Diego, CA, United States) and compared with controls (Varier and Sumathi, 2019).

### Cell Cycle Measurement

Cells were seeded in a 6-well culture plate at a density of 1 × 10^6^ cells/ml in 2 ml medium and were treated with 3g at different concentrations (0.3, 0.5, and 1 µM) for 24 and 48 h. After the incubation period, the cells were harvested and transferred into a sterile centrifuge tube for cell cycle analysis (Gajendran et al., 2020b). Cells were washed with pre-cool PBS and suspended in 70% ice ethanol, incubated for 4 h at 4°C, and preserved in a refrigerator at −20°C overnight. To remove the stationary liquid, the cells were centrifuged and washed twice with cold PBS. Then, 500 µL mix dye solution (RNaseA 100 μg/mL, PI 50 μg/mL, and Triton X-100 0.2%) were added into each tube, gently mixed, and incubated for 10 min at room temperature in the dark. Before analysis by flow cytometry, the cells were washed with cold PBS, and 200 µl suspensions were used for analysis by a NovoCyte flow cytometer (ACEA Biosciences, Inc. San Diego, CA, United States).

### Measurement of Mitochondrial Membrane Potential (∆Ψm)

Cells (1 × 10^6^) were seeded in a 3 ml medium with different concentrations of 3g (0.3, 0.5, and 1 µM) to detect the change in the membrane potential of treated cells concerning control cells. After being treated with 3g for 48 h, the cells were preincubated with JC-1 working solution for 20 min at 37°C, 5% CO_2_. After incubation, the dye was removed, and the cells were washed two times with JC-1 (Ma et al., 2021) and investigated using a Nikon fluorescent microscope.

### Hoechst 33258 Staining

K562 cells (1 × 10^6^) were seeded into a 6-well plate and treated with different concentrations of 3g (0.3, 0.5, and 1 µM) for 72 h (Ma et al., 2021). Cells were consequently collected, rinsed twice with PBS, and stained with Hoechst 33258 (Beyotime, Jiangsu, China) for 10 min and examined by using a Nikon fluorescence microscope.

### Western Blot Analysis

Cells were treated with 3g in different concentrations (0.3, 0.5, and 1 µM) for 24 h, cells were extracted, and total protein was collected from cell lysis buffer. Protein concentration was determined by the BCA test kit (Solarbio Life Sciences, China), and proteins were separated by 10% SDS-PAGE, then blotted onto the PVDF membrane (0.22 µm, Merck KGaA, Germany). The membranes were incubated in solution with 5% milk (dissolved in TBST) at room temperature for 1 h and probed with primary antibodies and GAPDH at 4°C overnight. After TBST washing, the membrane was developed with a secondary antibody (Hu et al., 2021). The antibodies were diluted according to the manufacturer’s instructions. Last, immunoreactive protein signals were spotted by the Odyssey Infrared Imaging System. GAPDH served as an internal loading control.

### AutoDock Analysis

The molecular docking simulation was performed *via* the AutoDock Vina program (Morris et al., 2009; Varier et al., 2017). ERK (PDB code: 5V60) interaction with U0126 (PubChem ID: 3006531) and 3g were carefully analyzed.

### Statistical Analysis

All statistical analyses by two-way ANOVA test were performed, followed by Tukey’s *post hoc* test analysis by GraphPad Prism 8 software (San Diego, CA, United States). All the experiments were organized in triplicates. The data were expressed as mean ± SD with significant *p* values (*p < 0.05, **p < 0.01, ***p < 0.001, and ****p < 0.0001).

## Results

### Chemistry

**FIGURE 1:**
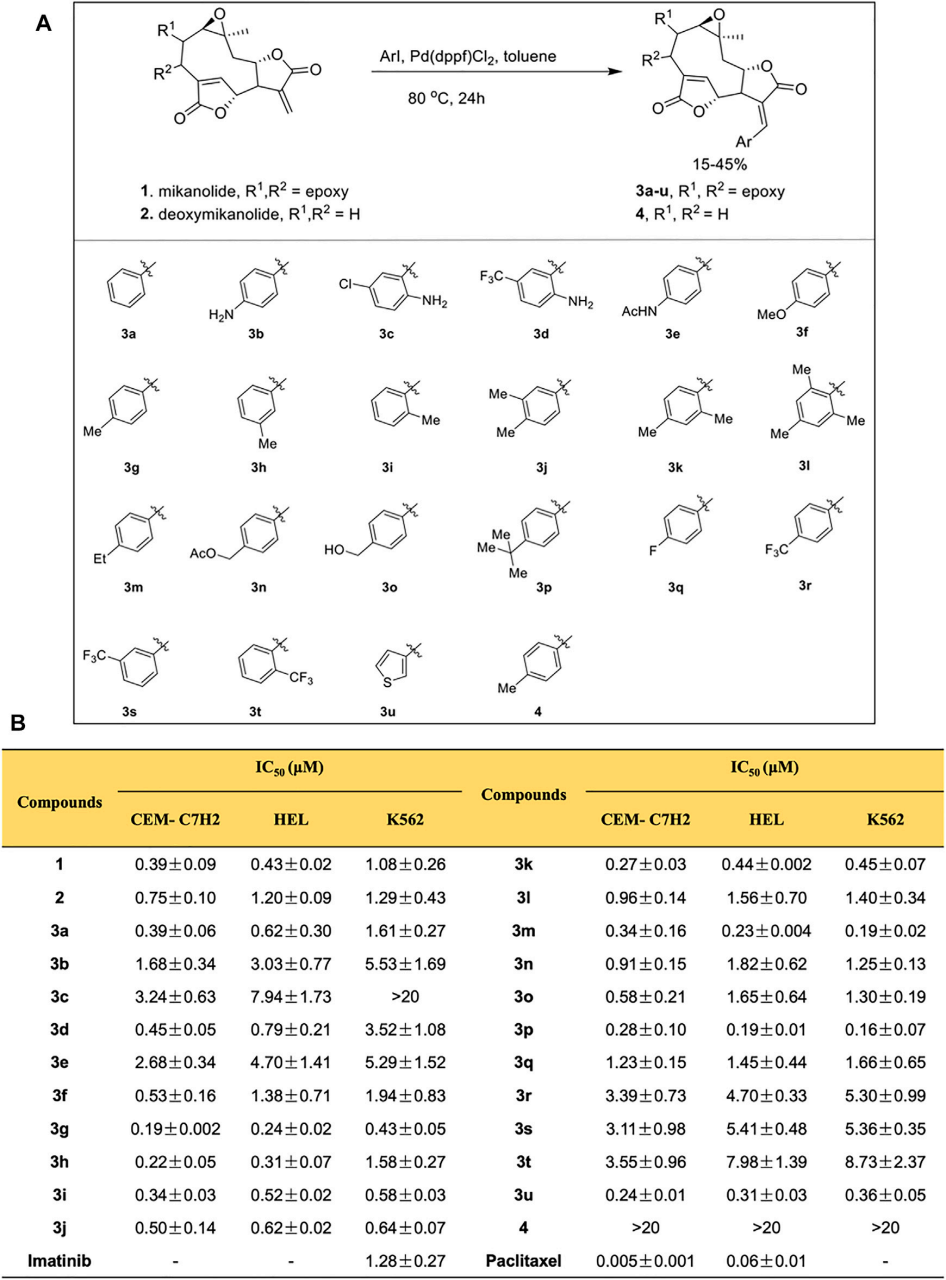
Mikanolide derivatives offer antileukemic action. **(A)** Schematic diagram explaining the procedure of synthesis of mikanolide derivatives. **(B)** Cytotoxicity (IC_50_) of mikanolide derivatives against indicated cancer cells. Data represented as means ± SD of three independent experiments.

Mikanolide and deoxymikanolide were isolated according to the reported procedures (Li et al., 2013; Li et al., 2013), and 22 novel E-olefinic coupling products of mikanolides (3a-u and 4) were prepared in 15–45% yields under heck reaction conditions utilizing palladium ferrocene [Pd (dppf)Cl_2_] as a catalyst in toluene and heating the mixture with an appropriate iodo-aromatic or iodo-heteroaromatic compound in the presence of di-isopropylethyl-amine (DIPEA) as the base (Figure 1A; Supplementary Material).

Compound 3a (1aR,1bR,2aR,6R,6aR,9aS,10aS,Z)-7-[(E)-benzylidene]-10a-methyl-1a,1b,2a,6a,7,9a,10,10a-octahydro-4H-3,6-(metheno) furo [3,2-c] bis (oxireno) [2,3-f:2',3'-h] [1] oxacycloundecine-4,8 (6H)-dione: 149.5 mg; isolated yield 39.5%; white solid; IR (KBr): 2,926.49, 2,359.84, 1,755.88, 1,645.58, 1,645.58, 1,195.91, 1,079.00, 1,021.15, 804.95, 696.93, and 472.58 cm^−1^; ^1^H NMR (600 MHz, CDCl_3_): δ 7.87 (d, J = 3.0 Hz, 1H), 7.62 (d, J = 7.8 Hz, 2H), 7.52 (t, J = 7.5 Hz, 2H), 7.47 (t, J = 7.5 Hz, 1H), 7.11 (s, 1H), 5.20 (s, 1H), 4.37 (m, 1H), 4.05 (s, 1H), 3.83 (m, 1H), 3.38 (d, J = 3.6 Hz, 1H), 2.94 (s, 1H), 2.21 (m, 2H), and 1.24 (m, 3H) ppm; ^13^C NMR (125 MHz, CDCl_3_): δ 170.0, 169.0, 146.2, 141.3, 131.8, 131.4, 130.9, 130.5, 129.0, 124.6, 79.7, 78.5, 58.4, 57.8, 54.9, 51.0, 50.6, 43.4, and 21.1 ppm. HRMS calculated for C_21_H_19_NO_6_, (M + H)+: 367.1176 found 367.1168.

Compound 3b (1aR,1bR,2aR,6R,6aR,9aS,10aS,Z)-7-[(E)-4-aminobenzylidene]-10a-methyl-1a,1b,2a,6a,7,9a,10,10a-octahydro-4H-3,6-(metheno) furo [3,2-c] bis (oxireno) [2,3-f:2',3'-h] [1] oxacycloundecine-4,8 (6H)-dione: 161.6 mg; isolated yield 41.0%; light yellow solid; IR (KBr): 3,473.59, 3,365.14, 3,239.04, 2,935.41, 2,360.15, 1744.47, 1,519.48, 1,365.13, 1,309.06, 1,261.56, 1,199.75, 1,177.24, 1,106.54, 1,078.05, 1,035.83, 1,012.46, 928.06, 831.10, and 626.06 cm^−1^; ^1^H NMR (600 MHz, CDCl_3_): δ 7.67 (d, J = 3.6 Hz, 1H), 7.47 (d, J = 8.4 Hz, 2H), 7.17 (s, 1H), 6.75 (d, J = 8.4 Hz, 2H), 5.30 (s, 1H), 4.59 (d, J = 7.8 Hz, 1H), 3.88 (dd, J = 10.8, 3.0 Hz, 1H), 2.70 (m, 2H), 2.56 (m, 1H), 2.18 (s, 2H), and 1.32 (s, 3H) ppm; ^13^C NMR (125 MHz, CDCl_3_): δ 171.6, 170.3, 149.3, 147.6, 140.9, 133.1, 133.0, 121.8, 118.7, 114.7, 78.5, 78.2, 61.8, 57.0, 50.5, 44.0, 22.8, 22.3, and 19.9 ppm. HRMS calculated for C_21_H_20_NO_6_, (M + H)+: 382.1285 found 382.1275.

Compound 3c (1aR,1bR,2aR,6R,6aR,9aS,10aS,Z)-7-[(E)-2-amino-5-chlorobenzylidene]-10a-methyl-1a,1b,2a,6a,7,9a,10,10a-octahydro-4H-3,6-(metheno) furo [3,2-c] bis (oxireno) [2,3-f:2',3'-h] [1] oxacycloundecine-4,8 (6H)-dione: 180.3 mg; isolated yield 42.0%; light yellow solid: IR (KBr): 3,367.06, 2,935.41, 2,360.36, 1748.95, 1,635.78, 1,487.85, 1,418.17, 1,364.27, 1,300.64, 1,265.09, 1,228.68, 1,188.36, 1,105.23, 1,035.56, 1,012.47, 930.06, and 635.04 cm^−1^; ^1^H NMR (600 MHz, CDCl_3_): δ 7.70 (d, J = 3.6 Hz, 1H), 7.36 (d, J = 1.8 Hz, 1H), 7.20 (dd, J = 10.8, 2.4 Hz, 1H), 7.09 (s, 1H), 6.73 (d, J = 9.0 Hz, 1H), 5.05 (s, 1H), 4.58 (m, 1H), 3.88 (m, 1H), 2.69 (m, 2H), 2.52 (m, 1H), 2.20 (m, 2H), and 1.32 (s, 3H) ppm; ^13^C NMR (125 MHz, CDCl_3_): δ 171.3, 169.3, 146.9, 144.0, 134.2, 133.3, 131.8, 128.6, 126.3, 123.6, 118.4, 117.9, 78.4, 77.9, 61.8, 56.9, 50.5, 44.0, 22.7, 22.1, and 20.0 ppm. HRMS calculated for C_21_H_19_C_l_NO_6_, (M + H)+: 416.0895 found 416.0886.

Compound 3 d (1aR,1bR,2aR,6R,6aR,9aS,10aS,Z)-7-[(E)-2-amino-5-(trifluoromethyl)benzylidene]-10a-methyl-1a,1b,2a,6a,7,9a,10,10a-octahydro-4H-3,6-(metheno) furo [3,2-c] bis (oxireno) [2,3-f:2',3'-h] [1] oxacycloundecine-4,8 (6H)-dione: 180.3 mg; isolated yield 42.0%; white solid; IR (KBr): 3,400.39, 2,359.86, 1775.83, 1,634.46, 1,510.61, 1,333.31, 1,266.66, 1,230.21, 1,111.72, 1,017.45, 843.14, 802.66, 730.89, and 602.09 cm^−1^; ^1^H NMR (600 MHz, DMSO-d6): δ 7.68 (s, 1H), 7.54 (m, 2H), 7.38 (dd, J = 10.2, 1.8Hz, 1H), 6.82 (d, J = 8.4 Hz, 1H), 6.16 (s, 2H), 5.01 (s, 1H), 4.77 (m, 1H), 4.00 (m, 1H), 3.93 (d, J = 3.0 Hz, 1H), 3.38 (d, J = 3.6 Hz, 1H), 3.20 (s, 1H), 2.30 (t, J = 12.0 Hz, 1H), 1.84 (dd, J = 17.4, 4.2 Hz, 1H), and 1.05 (s, 3H) ppm, ^13^C NMR (125 MHz, DMSO-d6): δ 170.4, 169.2, 150.8, 149.6, 133.4, 128.7, 128.4, 127.6, 127.5, 125.9, 124.1, 115.6, 114.9, 81.1, 77.4, 57.5, 57.3, 54.9, 50.2, 48.9, 42.1, and 20.6 ppm; ^19^F NMR (125 MHz, DMSO-d6): 59.2 (3) ppm. HRMS calculated for C_22_H_19_F_3_NO_6_, (M + H)+: 450.1159 found 450.1160.

Compound 3e N-{4-(E)-[(1aR,1bR,2aR,6R,6aR,9aS,10aS,Z)-10a-methyl-4,8-dioxo-1a,1b,2a,6,6a,9a,10,10a-octahydro-4H-3,6-(metheno) furo [3,2-c] bis (oxireno) [2,3-f:2',3'-h] [1] oxacycloundecin-7 (8H)-ylidene)methyl] phenyl} acetamide: 155.3 mg; isolated yield 35.5%; light yellow solid: IR (KBr): 3,577.39, 2,359.53, 1769.28, 1748.68, 1,683.73, 1,635.05, 1,592.76, 1,514.66, 1,415.59, 1,371.33, 1,313.69, 1,259.48, 1,199.65, 1,179.40, 1,079.17, 1,024.75, 840.01, 803.69, and 679.07 cm^−1^; ^1^H NMR (600 MHz, DMSO-d6): δ 10.2 (s, 1H), 7.69 (m, 4H), 7.56 (t, J = 6.0,Hz, 2H), 5.13 (s, 1H), 4.73 (m, 1H), 4.11 (t, J = 3.6 Hz, 1H), 3.98 (d, J = 3.0 Hz, 1H), 3.40 (d, J = 3.0 Hz, 1H), 3.16 (s, 1H), 2.24 (t, J = 12.6 Hz, 1H), 2.08 (d, J = 6.0 Hz, 3H), 1.84 (dd, J = 16.8, 3.0 Hz, 1H), and 1.07 (s, 3H) ppm; ^13^C NMR (125 MHz, DMSO-d6): δ 170.7, 169.6, 168.7, 150.0, 141.0, 137.6, 131.4, 128.6, 127.0, 125.8, 118.3, 81.2, 77.8, 57.7, 57.4, 54.7, 50.3, 48.8, 42.0, 24.2, and 20.5 ppm. HRMS calculated for C_23_H_22_NO_7_, (M + H)+: 424.1391 found 424.1393.

Compound 3f (1aR,1bR,2aR,6R,6aR,9aS,10aS,Z)-7-[(E)-4-methoxybenzylidene]-10a-methyl-1a,1b,2a,6a,7,9a,10,10a-octahydro-4H-3,6-(metheno) furo [3,2-c] bis (oxireno) [2,3-f:2',3'-h] [1] oxacycloundecine-4,8 (6H)-dione: 163.8 mg; isolated yield 41.4%; white solid; IR (KBr): 2,358.91, 1751.23, 1,644.06, 1,604.30, 1,514.57, 1,307.14, 1,216.08, 1,199.72, 1,176.07, 1,021.69, and 805.33 cm^−1^; ^1^H NMR (600 MHz, CDCl_3_): δ 7.78 (d, J = 3.0 Hz, 1H), 7.62 (d, J = 8.4 Hz, 2H), 7.16 (s, 1H), 7.03 (d, J = 8.4 Hz, 2H), 5.28 (d, J = 1.8 Hz, 1H), 4.38 (m, 1H), 4.06 (d, J = 3.0 Hz, 1H), 3.88 (s, 3H), 3.80 (m, 1H), 3.38 (d, J = 3.0 Hz, 1H), 2.95 (s, 1H), 2.22 (m, 2H), and 1.25 (s, 3H) ppm; ^13^C NMR (125 MHz, CDCl_3_): δ 170.1, 169.5, 161.8, 146.6, 141.1, 132.7, 131.2, 124.3, 121.5, 114.5, 79.7, 78.5, 58.3, 57.9, 55.5, 54.9, 51.0, 50.7, 43.3, and 21.1 ppm. HRMS calculated for C_22_H_21_O_7_, (M + H)+: 397.1282 found 397.1283.

Compound 3g (1aR,1bR,2aR,6R,6aR,9aS, 10aS,Z)-10a-methyl-7-[(E)-4-methylbenzylidene]-1a,1b,2a,6a,7,9a, 10,10a-octahydro-4H-3,6-(metheno) furo [3,2-c] bis (oxireno) [2,3-f:2',3'-h] [1] oxacycloundecine-4,8(6H)-dione: 78.0 mg; isolated yield 39.7%; white solid; IR (KBr):2,959.55, 2,359.98, 1764.47, 1,651.30, 1,608.26, 1,510.49, 1,353.13, 1,261.73, 1,197.54, 1,081.93, 1,022.75, 896.31, 736.61, and 677.40 cm^−1^; ^1^H NMR (600 MHz, CDCl_3_): δ 7.82 (d, J = 3.6 Hz, 1H), 7.53 (d, J = 7.8 Hz, 2H), 7.33 (d, J = 8.4 Hz, 2H), 7.13 (s, 1H), 5.24 (d, J = 1.8 Hz, 2H), 4.37 (m, 1H), 4.05 (d, J = 2.4 Hz, 1H), 3.82 (m, 1H), 3.38 (d, J = 3.6 Hz, 1H), 2.94 (s, 1H), 2.42 (s, 3H), 2.22 (m, 2H), and 1.25 (s, 3H) ppm; ^13^C NMR (125 MHz, CDCl_3_): δ 170.0, 169.3, 146.6, 141.6, 141.4, 131.3, 130.6, 129.7, 128.9, 123.3, 79.7, 78.5, 58.3, 57.9, 54.9, 51.0, 50.7, 43.3, 21.6, and 21.1 ppm. HRMS calculated for C_22_H_20_O_6_Na, (M + Na)+: 403.1152 found 403.1144.

Compound 3h (1aR,1bR,2aR,6R,6aR,9aS, 10aS,Z)-10a-methyl-7-[(E)-3-methylbenzylidene]-1a,1b,2a,6a,7,9a, 10,10a-octahydro-4H-3,6-(metheno) furo [3,2-c] bis (oxireno) [2,3-f:2',3'-h] [1] oxacycloundecine-4,8 (6H)-dione: 149.4 mg; isolated yield 38.0%; white solid; IR (KBr): 2,929.12, 2,359.41, 1764.42, 1,372.25, 1,347.99, 1,264.94, 1,209.79, 1,165.25, 1,084.03, 1,022.65, 892.60, 790.61, and 697.10 cm^−1^; ^1^H NMR (600 MHz, DMSO-d6): δ 7.84 (d, J = 3.6 Hz, 1H), 7.46 (s, 1H), 7.41 (d, J = 5.4 Hz, 2H), 7.28 (m, 1H), 7.10 (s, 1H), 5.20 (d, J = 1.8 Hz, 2H), 4.37 (m, 1H), 4.05 (d, J = 3.0 Hz, 1H), 3.82 (m, 1H), 3.38 (d, J = 3.0 Hz, 1H), 2.94 (s, 1H), 2.46 (s, 3H), 2.18 (m, 2H), and 1.25 (s, 3H) ppm; ^13^C NMR (125 MHz, DMSO-d6): δ 169.9, 169.1, 146.2, 141.5, 138.8, 131.7, 131.6, 131.3, 131.0, 128.8, 127.7, 124.3, 79.6, 78.4, 58.3, 57.8, 54.9, 51.0, 50.7, 43.4, 21.1, and 18.4 ppm. HRMS calculated for C_22_H_21_O_6_, (M + H)+: 381.1333 found 381.1323.

Compound 3i (1aR,1bR,2aR,6R,6aR,9aS,10aS,Z)-10a-methyl-7-[(E)-2-methylbenzylidene] 1a,1b,2a,6a,7,9a,10,10a-octahydro-4H-3,6-(metheno) furo [3,2-c] bis (oxireno) [2,3-f:2',3'-h] [1] oxacycloundecine-4,8 (6H)-dione: 143.5 mg; isolated yield 36.5%; white solid; IR (KBr): 2,920.46, 2,359.93, 1,743.78, 1,662.12, 1,457.26, 1,376.24, 1,349.31, 1,262.43, 1,212.20, 1,083.32, 1,024.69, 804.54, 755.47, and 678.90 cm^−1^; ^1^H NMR (600 MHz, CDCl_3_): δ 7.68 (d, J = 3.6 Hz, 1H), 7.63 (d, J = 7.8 Hz, 1H), 7.47 (d, J = 1.2 Hz, 1H), 7.32 (m, 1H), 7.29 (m, 2H), 4.96 (s, 1H), 4.74 (m, 1H), 4.02 (m, 1H), 3.93 (d, J = 3.6 Hz, 1H), 3.37 (dd, J = 4.8, 1.2 Hz, 1H), 3.18 (s, 1H), 2.35 (s, 3H), 2.26 (t, J = 12.3 Hz, 1H), 1.85 (dd, J = 16.8, 3.6 Hz, 1H), and 1.02 (s, 3H) ppm; ^13^C NMR (125 MHz, CDCl_3_): δ 170.6, 169.2, 149.7, 137.3, 136.3, 131.9, 129.8, 129.7, 129.3, 129.1, 128.6, 125.7, 81.4, 77.7, 57.5, 57.3, 54.8, 50.2, 48.4, 42.1, 20.6, and 19.6 ppm. HRMS calculated for C_22_H_21_O_6_, (M + H)+: 381.1333 found 381.1323.

Compound 3j (1aR,1bR,2aR,6R,6aR,9aS,10aS,Z)-7-[(E)-3,4-dimethylbenzylidene]-10a-methyl-1a,1b,2a,6a,7,9a,10,10a-octahydro-4H-3,6-(metheno) furo [3,2-c] bis (oxireno) [2,3-f:2',3'-h] [1] oxacycloundecine-4,8(6H)-dione: 144.3 mg; isolated yield 35.4%; white solid; IR (KBr): 2,918.19, 2,359.93, 1766.70, 1747.04, 1,652.51, 1,454.87, 1,373.63, 1,348.69, 1,265.66, 1,205.77, 1,163.64, 1,022.18, and 669.13 cm^−1^; ^1^H NMR (600 MHz, DMSO-d6): δ 7.55 (m, 3H), 7.44 (d, J = 7.8 Hz, 1H), 7.24 (d, J = 7.8 Hz, 1H), 5.10 (s, 1H), 4.74 (m, 1H), 4.10 (m, 1H), 3.97 (d, J = 3.0 Hz, 1H), 3.16 (s, 1H), 2.27 (d, J = 9.0 Hz, 6H), 1.82 (m, 2H), and 1.07 (s, 3H) ppm; ^13^C NMR (125 MHz, DMSO-d6): δ 170.6, 169.6, 149.9, 138.9, 138.0, 136.4, 131.4, 129.9, 129.6, 128.6, 128.2, 126.4, 81.1, 77.9, 57.7, 57.4, 50.4, 50.2, 48.9, 42.0, 20.6, 19.4, and 19.1 ppm. HRMS calculated for C_23_H_23_O_6_, (M + H)+: 395.1489 found 395.1491.

Compound 3k (1aR,1bR,2aR,6R,6aR,9aS,10aS,Z)-7-[(E)-2,4-dimethylbenzylidene]-10a-methyl-1a,1b,2a,6a,7,9a,10,10a-octahydro-4H-3,6-(metheno) furo [3,2-c] bis (oxireno) [2,3-f:2',3'-h] [1] oxacycloundecine-4,8(6H)-dione: 142.6 mg; isolated yield 35.0%; white solid; IR (KBr): 2,989.49, 2,360.13, 1740.93, 1,632.70, 1,612.44, 1,496.93, 1,450.06, 1,407.02, 1,365.09, 1,297.59, 1,263.76, 1,226.20, 1,201.65, 1,123.63, 1,072.60, 1,025.97, 894.11, 803.86, and 747.24 cm^−1^; ^1^H NMR (600 MHz, DMSO-d6): δ 7.64 (d, J = 3.6 Hz, 1H), 7.56 (d, J = 7.2 Hz, 1H), 7.49 (d, J = 1.2 Hz, 1H), 7.12 (m, 2H), 5.00 (t, J = 1.2 Hz, 1H), 4.73 (m, 1H), 4.05 (m, 1H), 3.94 (d, J = 3.0 Hz, 1H), 3.37 (d, J = 3.6 Hz, 1H), 3.17 (s, 1H), 2.32 (d, J = 5.4 Hz, 6H), 2.25 (m, 1H), 1.84 (dd, J = 17.4, 3.6 Hz, 1H), and 1.04 (s, 3H) ppm; ^13^C NMR (125 MHz, DMSO-d6): δ 170.7, 169.4, 149.8, 139.5, 137.5, 136.1, 130.7, 129.5, 128.9, 128.6, 127.9, 126.3, 81.3, 77.7, 57.5, 57.3, 54.8, 50.3, 48.5, 42.1, 21.0, 20.6, and 19.5 ppm. HRMS calculated for C_23_H_23_O_6_, (M + H)+: 395.1489 found 395.1491.

Compound 3l (1aR,1bR,2aR,6R,6aR,9aS,10aS,Z)-10a-methyl-7-[(E)-2,4,6-trimethylbenzylidene]-1a,1b,2a,6a,7,9a,10,10a-octahydro-4H-3,6-(metheno) furo [3,2-c] bis (oxireno) [2,3-f:2',3'-h] [1] oxacycloundecine-4,8 (6H)-dione: 164.6 mg; isolated yield 39.0%; white solid; IR (KBr): 2,970.53, 2,359.70, 1759.72, 1,668.57, 1,342.95, 1,225.26, 1,202.19, 1,080.08, 1,018.43, 903.49, 848.07, 803.99, and 678.79 cm^−1^; ^1^H NMR (600 MHz, DMSO-d6): δ 7.60 (d, J = 3.0 Hz, 1H), 7.43 (s, 1H), 6.97 (s, 1H), 6.87 (s, 1H), 4.73 (m, 2H), 3.88 (d, J = 3.0 Hz, 1H), 3.35 (d, J = 3.0 Hz, 1H), 3.24 (m, 1H), 3.20 (s, 1H), 2.33 (t, J = 12.3 Hz, 1H), 2.22 (m, 9H), 1.85 (dd, J = 17.4, 3.6 Hz, 1H), and 0.98 (s, 3H) ppm; ^13^C NMR (125 MHz, DMSO-d6): δ 170.6, 169.5, 149.8, 146.1, 137.9, 130.5, 129.9, 128.6, 127.9, 126.7, 81.1, 77.9, 57.6, 57.4, 54.7, 50.3, 48.8, 42.0, 28.1, 20.5, and 15.2 ppm. HRMS calculated for C_24_H_25_O_6_, (M + H)+: 409.1646 found 409.1647.

Compound 3m (1aR,1bR,2aR,6R,6aR,9aS,10aS,Z)-7-[(E)-4-ethylbenzylidene]-10a-methyl-1a,1b,2a,6a,7,9a,10,10a-octahydro-4H-3,6-(metheno) furo [3,2-c] bis (oxireno) [2,3-f:2',3'-h] [1] oxacycloundecine-4,8 (6H)-dione: 152.0 mg; isolated yield 37.3%; white solid; IR (KBr): 2,968.14, 2,360.02, 1741.34, 1,637.90, 1,606.81, 1,508.35, 1,420.09, 1,370.07, 1,260.38, 1,225.74, 1,200.81, 1,181.62, 1,073.20, 1,023.79, 896.50, 834.37, 796.24, and 679.52 cm^−1^; ^1^H NMR (600 MHz, DMSO-d6): δ 7.66 (d, J = 7.8 Hz, 2H), 7.60 (s, J = 3.6 Hz, 1H), 7.54 (s, 1H), 7.33 (d, J = 8.4 Hz, 2H), 5.13 (s, 1H), 4.73 (m, 1H), 4.12 (m, 1H), 3.98 (d, J = 3.0 Hz, 1H), 3.40 (d, J = 3.6 Hz, 1H), 3.16 (s, 1H), 2.67 (q, J = 7.6 Hz, 2H), 2.26 (t, J = 12.6 Hz, 1H), 1.84 (dd, J = 16.8, 3.6 Hz, 1H), 1.21 (t, J = 7.5 Hz, 3H), and 1.07 (s, 3H) ppm; ^13^C NMR (125 MHz, DMSO-d6): δ 170.6, 169.5, 149.8, 146.1, 137.8, 130.5, 129.9, 128.6, 127.9, 126.7, 81.1, 77.9, 57.6, 57.4, 54.7, 50.3, 48.8, 42.0, 28.1, 20.5, and 15.2 ppm. HRMS calculated for C_23_H_23_O_6_, (M + H)+: 395.1489 found 395.1491.

Compound 3n 4-{(E)-[(1aR,1bR,2aR,6R,6aR,9aS,10aS,Z)-10a-methyl-4,8-dioxo-1a,1b,2a,6,6a,9a, 10,10a-octahydro-4H-3,6-(metheno) furo [3,2-c] bis (oxireno) [2,3-f:2',3'-h] [1] oxacycloundecin-7 (8H)-ylidene]methyl}benzyl acetate: 140.4 mg; isolated yield 30.1%; white solid; IR (KBr): 3,409.25, 2,359.98, 1740.10, 1,635.00, 1,115.80, and 668.60 cm^−1^; ^1^H NMR (600 MHz, DMSO-d6): δ 7.74 (d, J = 8.4 Hz, 2H), 7.63 (s, J = 3.6 Hz, 1H), 7.53 (d, J = 1.2 Hz, 1H), 7.48 (d, J = 8.4 Hz, 2H), 5.14 (s, 2H), 5.11 (s, 1H), 4.75 (m, 1H), 4.15 (m, 1H), 3.98 (d, J = 3.6 Hz, 1H), 3.40 (dd, J = 4.2, 0.6 Hz, 1H), 3.17 (s, 1H), 2.25 (t, J = 12.3 Hz, 1H), 2.10 (s, 3H), 1.85 (dd, J = 16.8, 3.6 Hz, 1H), and 1.07 (s, 3H) ppm; ^13^C NMR (125 MHz, DMSO-d6): δ 171.1, 170.7, 169.9, 150.3, 138.6, 137.7, 132.6, 130.9, 129.1, 128.5, 128.2, 81.7, 78.4, 65.5, 58.1, 57.8, 55.2, 50.8, 49.1, 42.5, 21.2, and 20.0 ppm. HRMS calculated for C_24_H_23_O_8_, (M + H)+: 439.1387 found 439.1389.

Compound 3o (1aR,1bR,2aR,6R,6aR,9aS,10aS,Z)-7-[(E)-4-(hydroxymethyl)benzylidene]-10a-methyl-1a,1b,2a,6a,7,9a,10,10a-octahydro-4H-3,6-(metheno) furo [3,2-c] bis (oxireno) [2,3-f:2',3'-h] [1] oxacycloundecine-4,8 (6H)-dione: 154.8 mg; isolated yield 37.8%; white solid; IR (KBr): 3,123.86, 2,975.22, 2,359.69, 1753.75, 1,644.59, 1,519.60, 1,376.80, 1,338.73, 1,257.45, 1,215.98, 1,167.84, 1,071.03, 1,020.90, 800.01, 738.41, and 645.26 cm^−1^; ^1^H NMR (600 MHz, DMSO-d6): δ 7.70 (d, J = 7.8 Hz, 2H), 7.62 (d, J = 3.6 Hz, 1H), 7.54 (s, 1H), 7.43 (d, J = 8.4 Hz, 2H), 5.32 (t, J = 5.4 Hz, 1H), 5.11 (s, 1H), 4.74 (m, 1H), 4.56 (d, J = 5.4 Hz, 2H), 4.14 (m, 1H), 3.97 (d, J = 3.0 Hz, 1H), 3.40 (d, J = 3.0 Hz, 1H), 3.33 (s, 1H), 2.25 (t, J = 12.3 Hz, 1H), 1.85 (dd, J = 16.8, 3.6 Hz, 1H), and 1.08 (s, 3H) ppm; ^13^C NMR (125 MHz, DMSO-d6): δ 170.6, 169.5, 149.8, 144.8, 137.8, 130.8, 130.3, 128.6, 127.1, 126.2, 81.1, 77.9, 62.6, 57.6, 57.4, 54.7, 50.3, 48.8, 42.0, and 20.5 ppm. HRMS calculated for C_22_H_20_O_7_Na, (M + Na)+: 419.1101 found 419.1094.

Compound 3p (1aR,1bR,2aR,6R,6aR,9aS, 10aS,Z)-7-[(E)-4-(tert-butyl)benzylidene]-10a-methyl-1a,1b,2a,6a,7,9a, 10,10a-octahydro-4H-3,6-(metheno) furo [3,2-c] bis (oxireno) [2,3-f:2',3'-h] [1] oxacycloundecine-4,8 (6H)-dione**:** 196.8 mg; isolated yield 45.1%; white solid; IR (KBr): 2,966.76, 2,359.91, 1741.72, 1,639.05, 1,607.81, 1,508.23, 1,458.24, 1,418.31, 1,364.24, 1,318.52, 1,287.23, 1,260.75, 1,188.70, 1,106.62, 1,072.97, 1,023.76, 895.73, 837.84, 795.91, and 752.83 cm^−1^; ^1^H NMR (600 MHz, DMSO-d6): δ 7.69 (d, J = 8.4 Hz, 2H), 7.60 (d, J = 3.6 Hz, 1H), 7.56 (s, 1H), 7.50 (d, J = 7.8 Hz, 2H), 5.18 (s, 1H), 4.74 (m, 1H), 4.01 (m, 1H), 3.98 (d, J = 3.0 Hz, 1H), 3.39 (d, J = 3.6 Hz, 1H), 3.17 (s, 1H), 2.27 (t, J = 12.6 Hz, 1H), 1.85 (dd, J = 16.8, 3.0 Hz, 1H), 1.31 (s, 9H), and 1.08 (s, 3H) ppm; ^13^C NMR (125 MHz, DMSO-d6): δ 170.7, 169.6, 152.9, 149.8, 137.7, 130.4, 129.7, 128.6, 126.6, 125.3, 81.2, 77.9, 57.6, 57.4, 54.7, 50.3, 48.8, 42.0, 34.7, 30.9, and 20.5 ppm. HRMS calculated for C_25_H_27_O_6_, (M + H)+: 423.1802 found 423.1804.

Compound 3q (1aR,1bR,2aR,6R,6aR,9aS, 10aS,Z)-7-[(E)-4-fluorobenzylidene]-10a-methyl-1a,1b,2a,6a,7,9a, 10,10a-octahydro-4H-3,6-(metheno) furo [3,2-c] bis (oxireno) [2,3-f:2',3'-h] [1] oxacycloundecine-4,8(6H)-dione:142.2 mg; isolated yield 35.8%; white solid; IR (KBr): 3,496.40, 2,977.92, 2,359.48, 1748.93, 1,655.73, 1,600.37, 1,508.59, 1,390.63, 1,349.85, 1,262.30, 1,218.84, 1,193.94, 1,159.43, 1,082.65, 1,022.77, 786.88, 737.41, and 678.17 cm^−1^; ^1^H NMR (600 MHz, DMSO-d6): δ 7.74 (t, J = 9.9 Hz, 2H), 7.58 (d, J = 4.8 Hz, 1H), 7.48 (s, 1H), 7.27 (t, J = 12.9 Hz, 2H), 5.03 (s, 1H), 4.70 (m, 1H), 4.06 (t, J = 5.4 Hz, 1H), 3.92 (d, J = 3.0 Hz, 1H), 3.33 (d, J = 4.8 Hz, 1H), 3.13 (s, 1H), 2.18 (t, J = 18.6 Hz, 1H), 1.80 (dd, J = 23.4, 4.2 Hz, 1H), and 1.08 (s, 3H) ppm; ^13^C NMR (125 MHz, DMSO-d6): δ 170.6, 169.3, 163.6, 162.0, 149.8, 136.7, 132.7, 129.2, 128.6, 128.0, 115.5, 115.4, 81.3, 77.8, 57.6, 57.3, 54.8, 50.3, 48.5, 42.0, and 20.5 ppm; ^19^F NMR (125 MHz, DMSO-d6): 110.3 ppm. HRMS calculated for C_21_H_18_FO_6_, (M + H)+: 385.1082 found 385.1083.

Compound 3r (1aR,1bR,2aR,6R,6aR,9aS,10aS,Z)-10a-methyl-7-[(E)-4-(trifluoromethyl)benzylidene]-1a,1b,2a,6a,7,9a,10,10a-octahydro-4H-3,6-(metheno) furo [3,2-c] bis (oxireno) [2,3-f:2',3'-h] [1] oxacycloundecine-4,8(6H)-dione (3r): 125.7 mg; isolated yield 28.0%; white solid; IR (KBr): 3,396.13, 2,922.14, 2,851.70, 2,359.82, 1748.13, 1,644.99, 1,420.16, 1,373.28, 1,324.64, 1,261.53, 1,225.56, 1,202.77, 1,166.46, 1,130.27, 1,067.68, 1,023.34, 840.75, 797.18, 744.85, 677.19, and 601.81 cm^−1^; ^1^H NMR (600 MHz, DMSO-d6): δ 7.93 (d, J = 7.8 Hz, 2H), 7.84 (d, J = 7.8 Hz, 2H), 7.71 (d, J = 3.0 Hz, 1H), 7.50 (s, 1H), 5.06 (s, 1H), 4.77 (m, 1H), 4.16 (t, J = 3.3 Hz, 1H), 3.96 (d, J = 2.4 Hz, 1H), 3.39 (d, J = 3.6 Hz, 1H), 3.20 (s, 1H), 2.25 (t, J = 12.3 Hz, 1H), 1.86 (dd, J = 16.2, 3.0 Hz, 1H), and 1.07 (s, 3H) ppm; ^13^C NMR (125 MHz, DMSO-d6): δ 170.6, 169.1, 149.8, 137.0, 135.8, 130.9, 130.8, 129.6, 128.7, 125.2, 123.2, 81.6, 77.9, 57.5, 57.3, 54.8, 50.3, 48.4, 42.0, 29.8, and 20.6 ppm; ^19^F NMR (125 MHz, DMSO-d6): 61.1 3) ppm. HRMS calculated for C22H_18_F_3_O_6_, (M + H)+: 435.1050 found 435.1051.

Compound 3s (1aR,1bR,2aR,6R,6aR,9aS,10aS,Z)-10a-methyl-7-[(E)-3-(trifluoromethyl)benzylidene]-1a,1b,2a,6a,7,9a,10,10a-octahydro-4H-3,6-(metheno) furo [3,2-c] bis (oxireno) [2,3-f:2',3'-h] [1] oxacycloundecine-4,8 (6H)-dione: 121.2 mg; isolated yield 27.0%; white solid; IR (KBr): 3,399.81, 2,918.51, 2,849.79, 2,360.10, 1748.57, 1,635.68, 1,558.17, 1,541.00, 1,507.72, 1,457.24, 1,130.23, and 668.88 cm^−1^; ^1^H NMR (600 MHz, DMSO-d6): δ 8.10 (s, 1H), 7.99 (d, J = 7.8 Hz, 1H), 7.79 (d, J = 7.2 Hz, 1H), 7.71 (m, 2H), 7.49 (s, 1H), 5.05 (s, 1H), 4.77 (m, 1H), 4.16 (m, 1H), 3.95 (s, 1H), 3.19 (s, 1H), 2.25 (t, J = 12.0 Hz, 1H), 1.86 (dd, J = 15.6, 3.0 Hz, 1H), and 1.07 (s, 3H) ppm; ^13^C NMR (125 MHz, DMSO-d6): δ 170.3, 169.1, 149.7, 135.9, 134.0, 133.8, 130.3, 129.4, 128.7, 126.7, 126.3, 124.9, 123.1, 81.4, 77.8, 57.5, 57.3, 54.8, 50.3, 48.4, 42.0, and 20.6 ppm; ^19^F NMR (125 MHz, DMSO-d6): 61.2 (3) ppm. HRMS calculated for C_22_H_18_F_3_O_6_, (M + H)+: 435.1050 found 435.1051.

Compound 3t (1aR,1bR,2aR,6R,6aR,9aS,10aS,Z)-10a-methyl-7-[(E)-2-(trifluoromethyl)benzylidene]-1a,1b,2a,6a,7,9a,10,10a-octahydro-4H-3,6-(metheno) furo [3,2-c] bis (oxireno) [2,3-f:2',3'-h] [1] oxacycloundecine-4,8 (6H)-dione: 67.3 mg; isolated yield 15.0%; white solid; IR (KBr): 3,566.03, 2,988.58, 2,360.09, 1773.14, 1,665.68, 1,488.49, 1,455.59, 1,352.39, 1,318.49, 1,229.70, 1,203.73, 1,165.16, 1,109.05, 1,021.95, 770.30, and 679.70 cm^−1^; ^1^H NMR (600 MHz, DMSO-d6): δ 7.95 (d, J = 7.8 Hz, 1H), 7.82 (m, 2H), 7.67 (m, 2H), 7.47 (d, J = 1.2 Hz, 1H), 4.89 (m, 1H), 4.81 (m, 1H), 4.00 (m, 1H), 3.91 (d, J = 3.6 Hz, 1H), 3.37 (dd, J = 4.8, 1.2 Hz, 1H), 3.18 (s, 1H), 2.25 (t, J = 12.3 Hz, 1H), 1.86 (dd, J = 17.4, 4.2 Hz, 1H), and 1.02 (s, 3H) ppm; ^13^C NMR (125 MHz, DMSO-d6): δ 170.4, 168.7, 149.8, 132.8, 132.7, 132.3, 131.2, 130.0, 128.7, 127.0, 125.9, 125.0, 123.7, 81.7, 77.6, 57.3, 57.2, 54.9, 50.1, 48.1, 42.0, and 20.6 ppm; ^19^F NMR (125 MHz, DMSO-d6): 58.9 (3) ppm. HRMS calculated for C_22_H_18_F_3_O_6_, (M + H)+: 435.1050 found 435.1051.

Compound 3u (1aR,1bR,2aR,3Z,6R,6aR,7E,9aS,10aS)-10a-methyl-7-(thiophen-3-ylmethylene)-1a,1b,2a,6a,7,9a,10,10a-octahydro-4H-3,6-(metheno) furo [3,2-c] bis (oxireno) [2,3-f:2',3'-h] [1] oxacycloundecine-4,8 (6H)-dione: 145.5 mg; isolated yield 37.8%; white solid; IR (KBr): 3,564.51, 2,932.29, 2,359.82, 1750.94, 1,654.39, 1,508.66, 1,418.21, 1,390.71, 1,262.38, 1,231.78, 1,198.39, 1,083.17, 1,022.15, 898.23, 805.15, and 677.67 cm^−1^; ^1^H NMR (600 MHz, DMSO-d6): δ 8.16 (d, J = 3.0 Hz, 1H), 7.71 (q, J = 2.6 Hz, 1H), 7.63 (d, J = 3.6 Hz, 1H), 7.60 (m, 2H), 5.27 (m, 1H), 4.73 (m, 1H), 3.99 (m, 2H), 3.40 (d, J = 3.6 Hz, 1H), 3.17 (s, 1H), 2.23 (t, J = 12.6 Hz, 1H), 1.83 (dd, J = 16.8, 3.6 Hz, 1H), and 1.08 (s, 3H) ppm; ^13^C NMR (125 MHz, DMSO-d6): δ 170.5, 169.7, 149.9, 134.4, 131.8, 131.1, 128.8, 128.6, 126.9, 125.6, 81.4, 77.8, 57.7, 57.4, 54.7, 50.3, 48.8, 42.0, and 20.5 ppm. HRMS calculated for C_19_ H_17_O_6_S, (M + H)+: 373.0740 found 373.0742.

Compound 4 (3aR,4R,9aR,10aS, 11aS,Z)-10a-methyl-3-[(E)-4-methylbenzylidene]-3a,4,8,9,9a,10a, 11,11a-octahydro-6H-4,7-(metheno) furo [3,2-c] oxireno [2,3-f] [1] oxacycloundecine-2,6 (3H)-dione: 110.0 mg; isolated yield 41.5%; white solid; IR (KBr): 3,083.01, 2,967.20, 2,359.81, 1749.00, 1,636.71, 1,603.52, 1,509.84, 1,452.20, 1,360.58, 1,314.44, 1,193.36, 1,178.89,1,106.03, 1,033.82, 1,012.46, 817.58, and 473.69 cm^−1^; ^1^H NMR (600 MHz, DMSO-d6): δ 7.73 (s, 1H), 7.64 (d, J = 8.4 Hz, 2H), 7.57 (d, J = 3.6 Hz, 1H), 7.33 (d, J = 7.8 Hz, 2H), 5.01 (s, 1H), 4.80 (m, 1H), 4.16 (m, 1H), 2.87 (dd, J = 13.8, 2.4 Hz, 1H), 2.54 (m, 1H), 2.45 (m, 1H), 2.36 (s, 3H), 2.22 (t, J = 12.6 Hz, 1H), 2.02 (m, 1H), 1.82 (dd, J = 17.4, 4.2 Hz, 1H), 1.29 (m, 1H), and 1.14 (s, 3H) ppm; ^13^C NMR (125 MHz, DMSO-d6): δ 172.5, 170.1, 151.7, 140.6, 137.6, 131.0, 130.9, 130.3, 129.8, 126.8, 80.0, 78.2, 61.0, 57.2, 49.1, 43.5, 23.3, 21.7, 21.5, and 20.0 ppm. HRMS calculated for C_22_ H_23_O_5_, (M + H)+: 367.1540 found 367.1541.

### Mikanolide Derivatives Possess Antileukemic Action

Using MTT assay, the cell viabilities of mikanolide derivatives were assessed. Paclitaxel and imatinib served as positive controls. Mikanolide derivatives were treated at different concentrations (0.0075–10 μmol/L) in CEM-C7H2, HEL, and K562 cells. The results exhibited significant antileukemic activity in the selected human leukemic cells. Notably, the IC_50_ value of mikanolide derivatives in 72 h showed good antiproliferative activity against the three leukemia cell lines (Figure 1B). However, among the 22 mikanolide derivatives, the compound 3g exhibited improved activity against the three leukemic cell lines.

### Compound 3g Potently Impedes Cell Proliferation in Leukemia Cells

**FIGURE 2:**
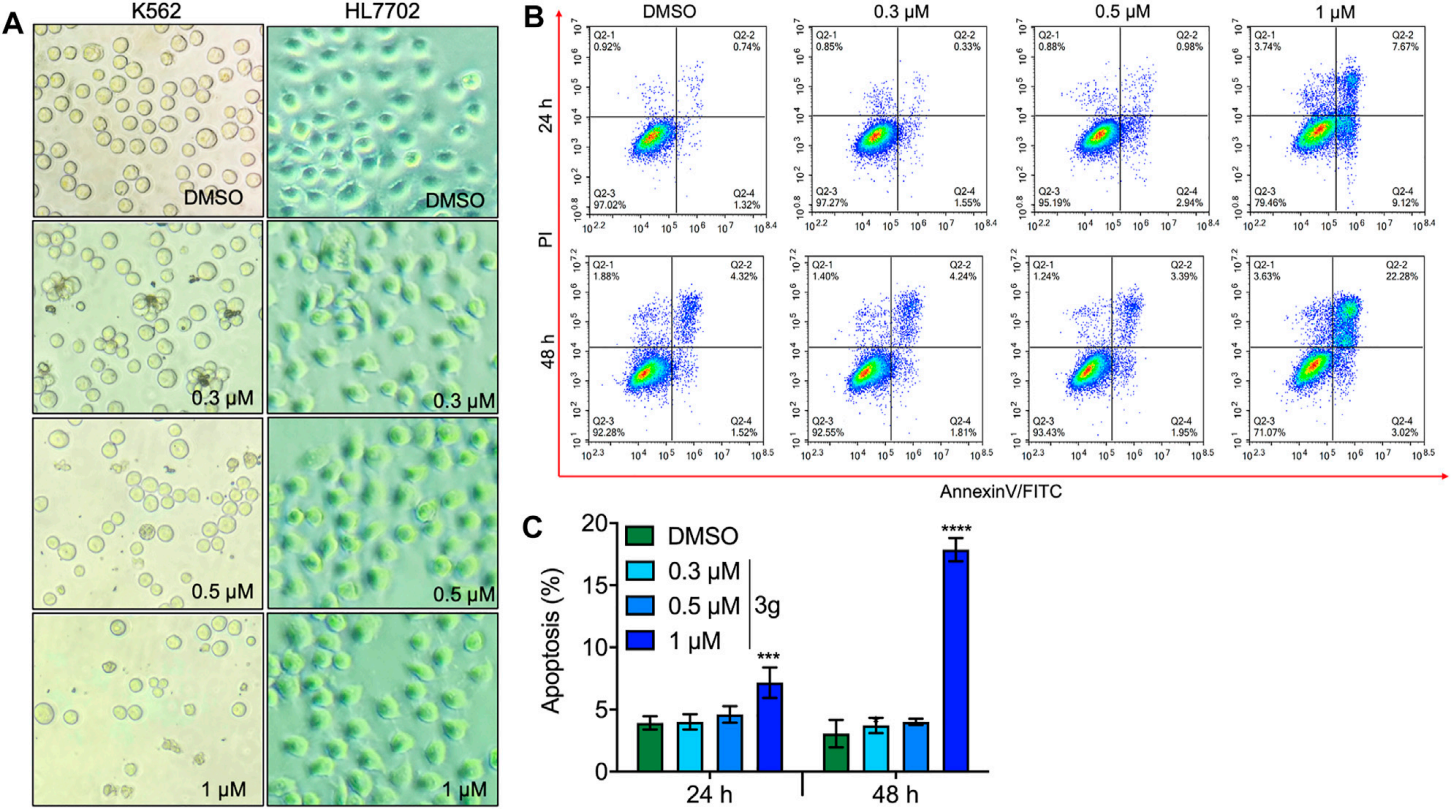
3g induces apoptosis in both dose- and time-dependent manners. **(A)** Dose-dependent cell morphology variations in K562 and normal hepatocyte cells at the indicated 3g concentrations (40X), for 72 h. **(B)** Flow cytometric analysis of apoptosis caused by indicated doses of 3g treatment at 24 and 48 h in K562 cells. **(C)** Densitometry of the apoptotic cells percentage at an indicated time interval of 3g incubation. Data are represented as mean ± SD (*n* = 3; ***p < 0.001 and ****p < 0.0001 vs. control).

The inhibition rate of 3g in K562 cells was effective in a dose- and time-dependent manner. Furthermore, 3g was assessed vs. normal hepatocyte cells, HL7702 (Figure 2A). When normal cells HL7702 were incubated with the 3g compound for 72 h, there were no variations in the cellular viability. But this compound showed a prominent decrease in cell viability to the human chronic myeloid leukemia cells K562 at the concentration of 0.43 ± 0.05 µM in 72 h. The results represented that 3g had a considerable antileukemic property in K562 cells (Figures 1B, 2A, Supplementary Figure S1), compared to the positive controls, imatinib (Supplementary Figure S2), and paclitaxel.

### 3g Triggers Apoptosis in Leukemia Cells

Different concentrations of 3g were treated in K562, HEL, and CEM-C7H2 cells after 24 and 48 h of incubation, to analyze the apoptotic effect using flow cytometry. The study revealed a significant apoptotic rate at higher concentrations in 24 and 48 h treatment of 3g (Figures 2B,C; Supplementary Figures S3, S4), compared to non-tumor lineage cells (Supplementary Figure S5).

### 3g Induces Cell Cycle Arrest in Leukemia Cells Both Dose- and Time-Dependently

**FIGURE 3:**
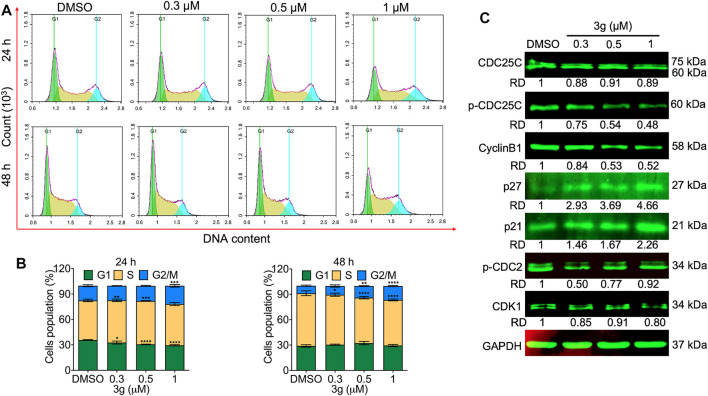
3g arrests the cell cycle in K562 cells. **(A)** Cell cycle arrest evaluation by flow cytometry after 24 and 48 h of 3g treatment. **(B)** Densitometry plots depicting the variations in the stages of cell cycle arrest. **(C)** Protein expression levels of indicated proteins after 48 h of 3g incubation. Data are represented as mean ± SD (*n* = 3; *p < 0.05, **p < 0.01, ***p < 0.001, and ****p < 0.0001 vs. control).

The cell cycle was analyzed to facilitate the apoptosis study. The study revealed a significant effect on the G_2_/M phase of the cell cycle, both dose- and time-dependently after 3g treatment in K562 cells (Figures 3A,B) and in the G_1_/G_2_ and S phases in HEL and CEM-C7H2 cells (Supplementary Figures S6, S7), respectively. There were no significant cell arrests in non-tumor lineage cells (Supplementary Figure S8). To reconfirm the hypothesis, Western blot was performed for cell cycle markers. There was a dose-dependent significant reduction in protein expressions of p-CDC25C and cyclin B1, with increased expression of P27, and P21, after 3g post-treatment (Figure 3C).

### 3g Engenders Mitochondrial and DNA Damage in K562 Cells

**FIGURE 4:**
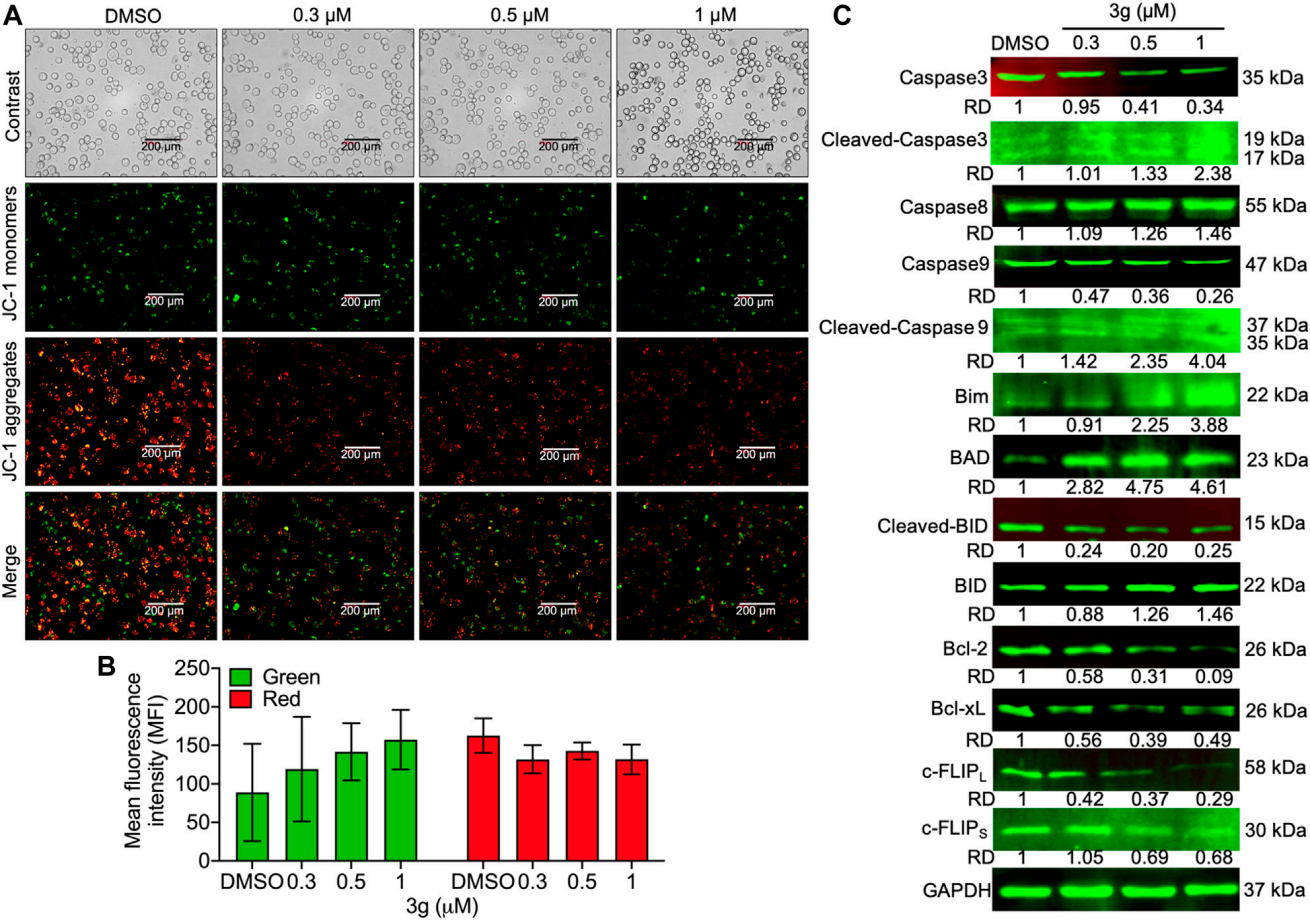
Mitochondrial damage after 3g treatment in K562 cells. **(A)** Photomicrograph of JC-1 monomers and aggregates after 3g incubation at 24 h. **(B)** Quantification of the red/green levels showing extent of mitochondrial damage. **(C)** Protein expression levels of indicated proteins at 48 h of 3g treatment.

**FIGURE 5:**
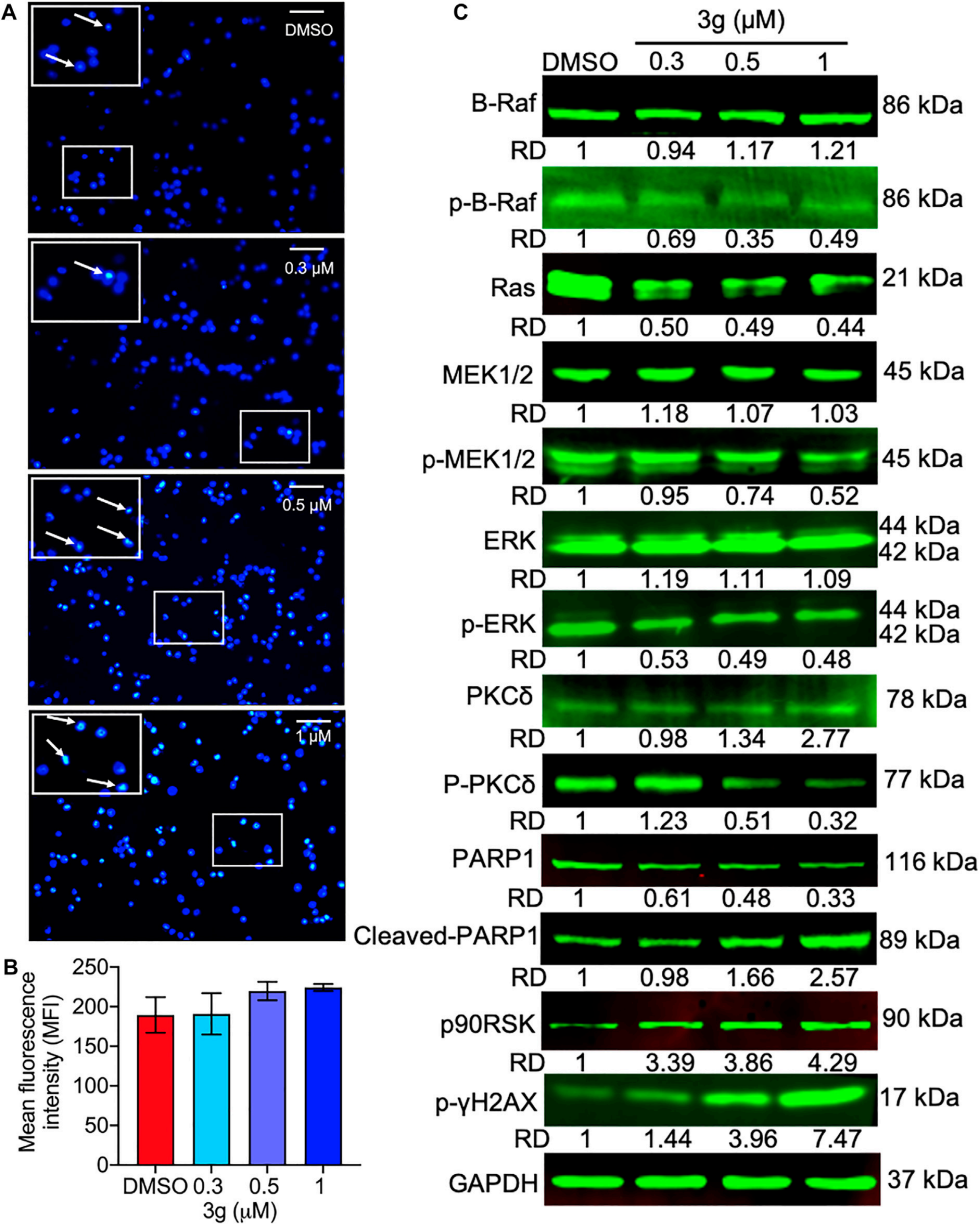
DNA damage and the Ras/Raf/Mek/Erk pathway alterations induced by 3g in K562 cells. **(A)** Photomicrograph of DNA damage (in boxes) at indicated concentrations of 3g treatment. **(B)** Quantification of the fluorescence levels showing extent of DNA damage. **(C)** Protein expression levels of indicated proteins after 3g treatment at 48 h.

To study whether the K562 cell’s mitochondria were affected by 3g treatment, we analyzed the loss of mitochondrial membrane potential (MMP). The results showed an increased green fluorescence after 3g treatment compared with control cells (Figures 4A,B). The reduced MMP suggests the damage to the chondriosome, by releasing cytochrome C and mitochondrial damage. Excitingly, the Western blot results further confirmed that the mitochondrial damage was accomplished by the 3g treatment in a dose-dependent manner, by decreased expression of Bcl-2, Bcl-XL, caspase 3, caspase 9, c-FLIP_L_, c-FLIP_S_, and cleaved BID. While, there was an increased expression of caspase 8, cleaved caspase 3, cleaved caspase 9, Bim, BAD, and BID proteins (Figure 4C). However, the Hoechst 33,258 staining revealed DNA damage, detected after K562 cells were treated with different concentrations of 3g. As shown in Figures 5A,B, the DNA damage levels were increased in a dose-dependent manner, suggesting cellular damage.

### 3g Selectively Targets the Ras/Raf/MEK/ERK Pathway in K562 Cells

**FIGURE 6:**
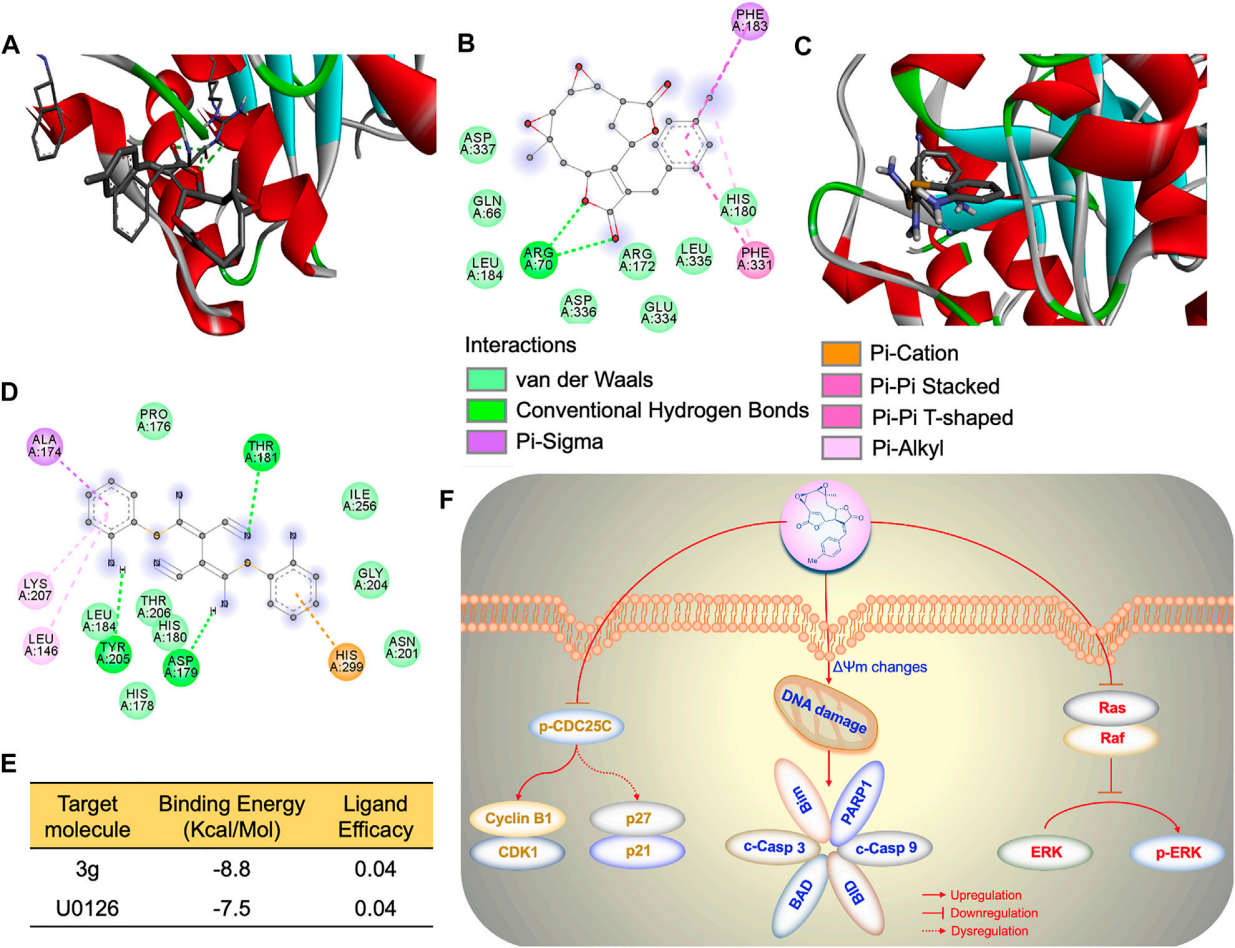
3g cause p-ERK inhibition. **(A)** AutoDock 3D image depicting the interactive site between U0126 and ERK. **(B)** AutoDock 2D amino acid interactive site image between U0126 and ERK. **(C)** AutoDock 3D image depicting the interactive site between 3g and ERK. **(D)** AutoDock 2D amino acid interactive site image between 3g and ERK. **(E)** Binding energy and ligand efficiency between ERK and ligands. **(F)** Molecular mechanism of 3g as an antileukemic agent.

The effect of 3g on Ras- and Raf-related molecules was analyzed by Western blot. The protein expressions of B-Raf, PKCδ, cleaved PARP1, p90RSK, and p-γH2AX were significantly increased. On the other hand, the expression levels of p-B-Raf, RAS, p-MEK1/2, p-PKCδ, PARP1, and p-ERK were significantly reduced, compared to the control. There were no variations in the expression levels of other molecules (Figure 5C). To further facilitate the specific molecular target of 3g, AutoDock was performed. The *in silico* study revealed that the 3g compound is a potent inhibitor of ERK (Figures 6A–D). With a considerable amount of binding energy and ligand efficiency such as U0126, 3g proved to inhibit functional p-ERK, which was elucidated by AutoDock (Figure 6E).

## Discussion

Leukemia is described by uncontrolled proliferation of blood cells, with limited cell death. The existence of leukemia is severe, generally affecting almost all ages of people. Thus, a strategy or drug of choice to treat the disease is in high demand. In this investigation, we prepared 22 novel mikanolide derivatives using heck reaction to study their antileukemic activities in human leukemia cell lines.

Biological agents and their derivatives are widely in the preclinical and clinical sectors for cancer chemotherapies. These agents have mainly been investigated for their properties to initiate apoptosis (Hanahan and Weinberg, 2000). Our investigation has indicated that mikanolide derivatives have cytotoxic effects on leukemic cells such as CEM-C7H2, HEL, and K562. The present study has proven that mikanolide derivatives have appropriate antileukemic activities. The selected mikanolide derivative compound, 3g inspired us to focus on K562 cells to predict the specific molecular antileukemic mechanism.

Apoptosis is a multi-stage process. Antiapoptosis is a key factor in many types of cancer. Protein kinases participate in the management of early stages of apoptosis by phosphorylating key apoptotic proteins or in later events by enacting downstream caspases. CDC25C can monitor G_2_/M phase progression and DNA damage repair. It is a specific phosphatase family cyclin that can trigger the cyclin B1/CDK1 complexes. These complexes can manage the cells inflowing with mitosis by regulating G_2_/M progression. They play as a checkpoint for protein regulation and DNA damage. The regulation of CDC25C is closely associated with tumorigenesis and is considered a possible target for cancer conduct (Liu et al., 2020). Likewise, dysfunction of p27 has been reported in human cancers, resulting from p27 phosphorylation-dependent protein degradation and cell cycle arrest (Sun et al., 2016). The p21 can regulate p53-dependent and independent cancer pathways, consenting to DNA repair and sponsoring tumorigenesis (Al Bitar and Gali-Muhtasib, 2019). In our investigation, a most effective mikanolide derivative, 3g arrested the K652 cells by entering the G_2_/M phase by increasing the expression of CDK1/cyclin B1. This blockage entry of the M phase by the mikanolide derivative is through inhibiting the activity of CDK1/cyclin B1 viz decreased phosphorylation of CDC25C or increased expression of p27 and p21 as explained earlier.

However, cancer cells have defective or uncontrolled cellular proliferation (Fulda et al., 2010; Indran et al., 2011). This apoptotic machinery may be either mitochondrial-dependent or -independent (Adams, 2003; Redza-Dutordoir and Averill-Bates, 2016). In our study, mikanolide derivative induced DNA and mitochondrial damage. So, disturbances in mitochondrial function can lead to cell impairment (Dai et al., 2014), 3g could downregulate cleaved caspase 3 and 9 and upregulate the Bim, BAD, and BID proteins, ensuring cellular mortality. Moreover, the cells treated with 3g decreased the expression of c-FLIP, Bcl-2, and Bcl-xL. c-FLIP regulates the apoptosis by attenuating autophagy by directly acting on the autophagy machinery by inhibiting autophagosome formation. The upregulation of c-FLIP has been found in various tumor types, and its silencing has been shown to restore apoptosis triggered by cytokines and various chemotherapeutic agents (Bagnoli et al., 2010). Similarly, 3g reduced the expression levels of c-FLIP dose-dependently. The nascent DNA responds to its damage by disrupting the histone phosphorylation in H2AX on its ser4 residue of C-terminus establishing γH2AX (Rogakou et al., 1998). This cellular response gets augmented about 20 min after the initiation of DNA damage (Redon et al., 2002). Thus, this molecule is considered as a powerful maker of DNA that breaks in the cancer cells experimentally. Likewise, the study elucidated that the drug 3g conferred DNA damage of K562 cell lysates by increasing the expression of γH2AX.

ERK is responsible for the broadcast of antiapoptotic signals from membrane-bound receptors (James et al., 2011). Chemotherapeutic drugs, frequently used in leukemia therapy, often inactivate this pathway. Inhibition of Ras (or Ras-related molecules), Raf, MEK, and ERK may prove useful in leukemia treatment. These observations have boosted the pharmaceutical industry to improve inhibitors that direct key factors of this pathway and are currently in clinical trials (McCubrey et al., 2007; McCubrey et al., 2008b; McCubrey et al., 2008b; Steelman et al., 2008; Steelman et al., 2010). The protein kinase C family of serine-threonine kinases is activated by diverse stimuli and participates in cellular processes such as growth, differentiation, and apoptosis (Abrams et al., 2010). The 90 kDa ribosomal S6 kinase (RSK) family of proteins is highly conserved Ser/Thr kinases that can regulate the downstream effectors of the ERK/MAPK trail (Hug and Sarre, 1993). Likewise, the 3g treatment, dose-dependently reduced the protein expression of p-ERK molecules, thus availing as a possible treatment for leukemia.

The MEK/ERK cascade sends signals to cell surface receptors and transcription factors that control gene expression. Moreover, additional signal transduction pathways usually interrelate with the Raf/MEK/ERK pathway to regulate its action, positively or negatively, by varying the phosphorylation of downstream targets (Anjum and Blenis, 2008), including phosphorylation of ERK (Chang et al., 2003; Ahearn et al., 2011). Thus, inhibitors of ERK pathway molecules are currently in clinical trials as anticancer agents (Knight and Irving, 2014). In line with it, in our study, mikanolide derivative 3g could significantly downregulate p-ERK. MEK is associated with apoptosis and cell cycle and the activation of P38, which could cause the p-CDC25C to dephosphorylate (Xiaofei and Kowalik, 2014), arresting K562 cells in the G_2_/M phase. These studies elucidated the inhibition of the pathway that could revive apoptosis, suggesting its capability to act as an apoptotic agent *via* p-ERK inhibition. Moreover, the *in silico* AutoDock studies discovered that ERK is inhibited by significant binding energies that might appeal for the downregulation and effect of 3g in the pathway.

## Conclusion

To conclude, our study revealed that mikanolide derivatives, especially 3g, could cross the K562 cells plasma membrane when administered an ideal dose. The 3g reduced the phosphorylation of CDC25C by significantly diminishing the expression of cyclin B1/CDK1, causing dysregulation of P27/P21, triggering cell cycle arrest, and leading to cellular apoptosis. Moreover, 3g could induce mitochondrial/DNA damage, leading to activation of caspase 3/9 and increased expression of cellular Bim, BAD, and BID proteins-mediated apoptosis. Similarly, 3g could inhibit phosphorylation of ERK, causing the dysregulation of the Ras/Raf/MEK/ERK pathway, promoting cell death of K562 cells (Figure 6F). These results thus suggest 3g as a novel potent chemotherapeutic agent for leukemia.

## Data Availability

The datasets presented in this study can be found in online repositories. The names of the repository/repositories and accession number(s) can be found in the article/Supplementary Material.
