# Supplementary material for: Design, Synthesis, and Antileukemic Evaluation of a Novel Mikanolide Derivative Through the Ras/Raf/MEK/ERK Pathway
Source: Front Pharmacol. 2022 May 20;13:809551. doi: 10.3389/fphar.2022.809551 (PMC9205396; doi:10.3389/fphar.2022.809551)
Supplement: Supplementary file 1 [file DataSheet2.PDF]

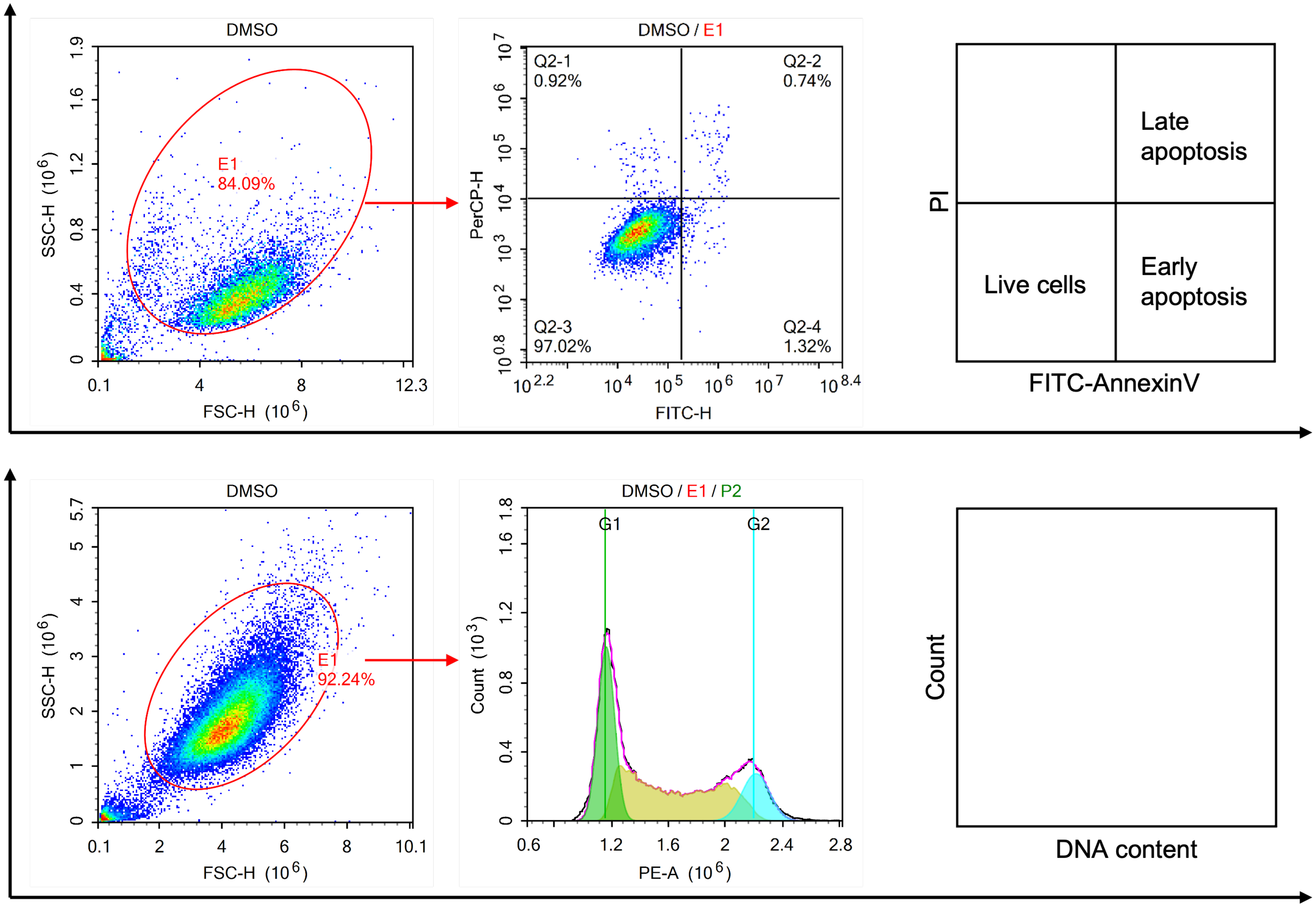

Flow cytometry gating strategy. A. Apoptosis analysis using FITC-AnnexinV/PI. Viable, late and early apoptotic fractions were identified using Annexin V. **B,** Cell cycle analysis using PI. The plot shows cells in G0/G1 phase (green), S phase (yellow) and G2/M phase (blue).
