# Supplementary figures and images for: Design, Synthesis, and Antileukemic Evaluation of a Novel Mikanolide Derivative Through the Ras/Raf/MEK/ERK Pathway
Source: Front Pharmacol. 2022 May 20;13:809551. doi: 10.3389/fphar.2022.809551 (PMC9205396; doi:10.3389/fphar.2022.809551)

Figure 3C

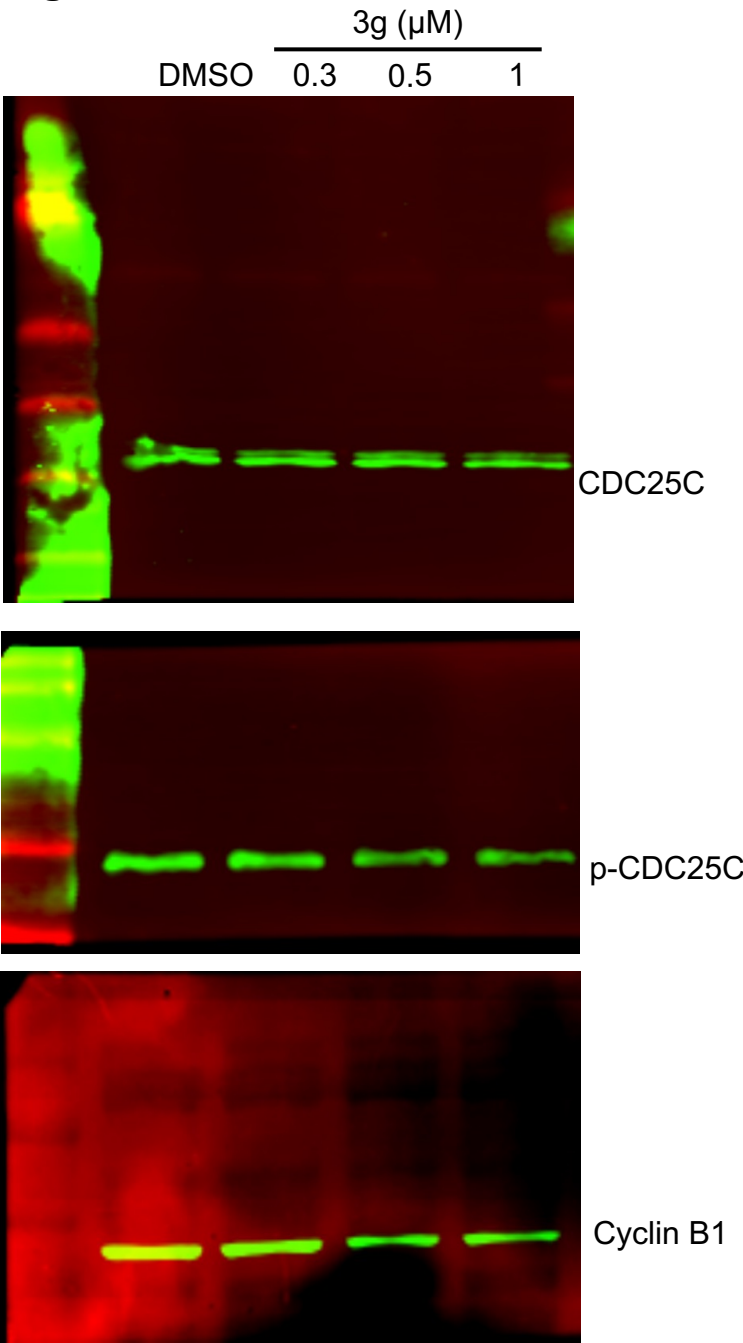

Uncropped full Western blot images

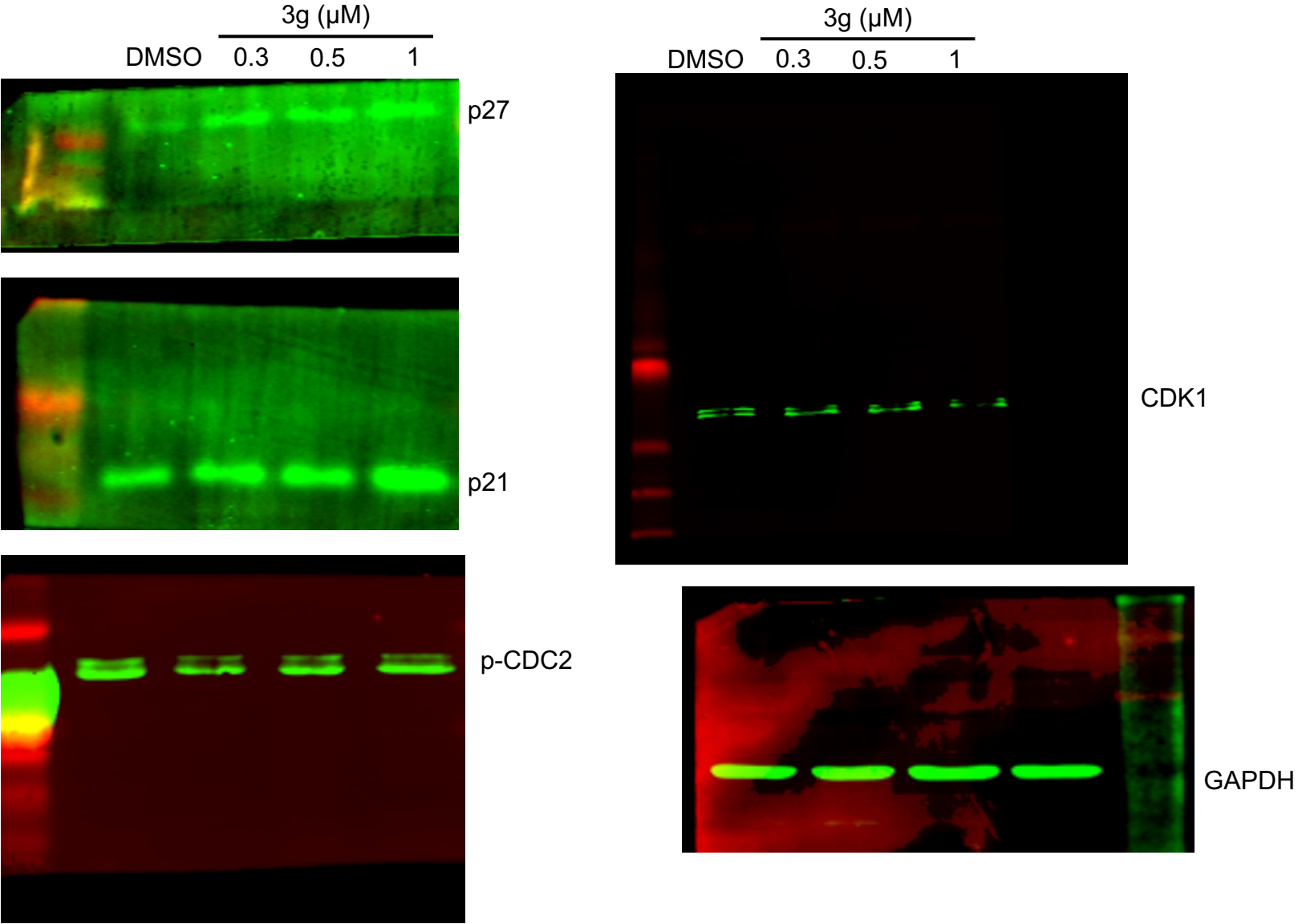

Figure 4B

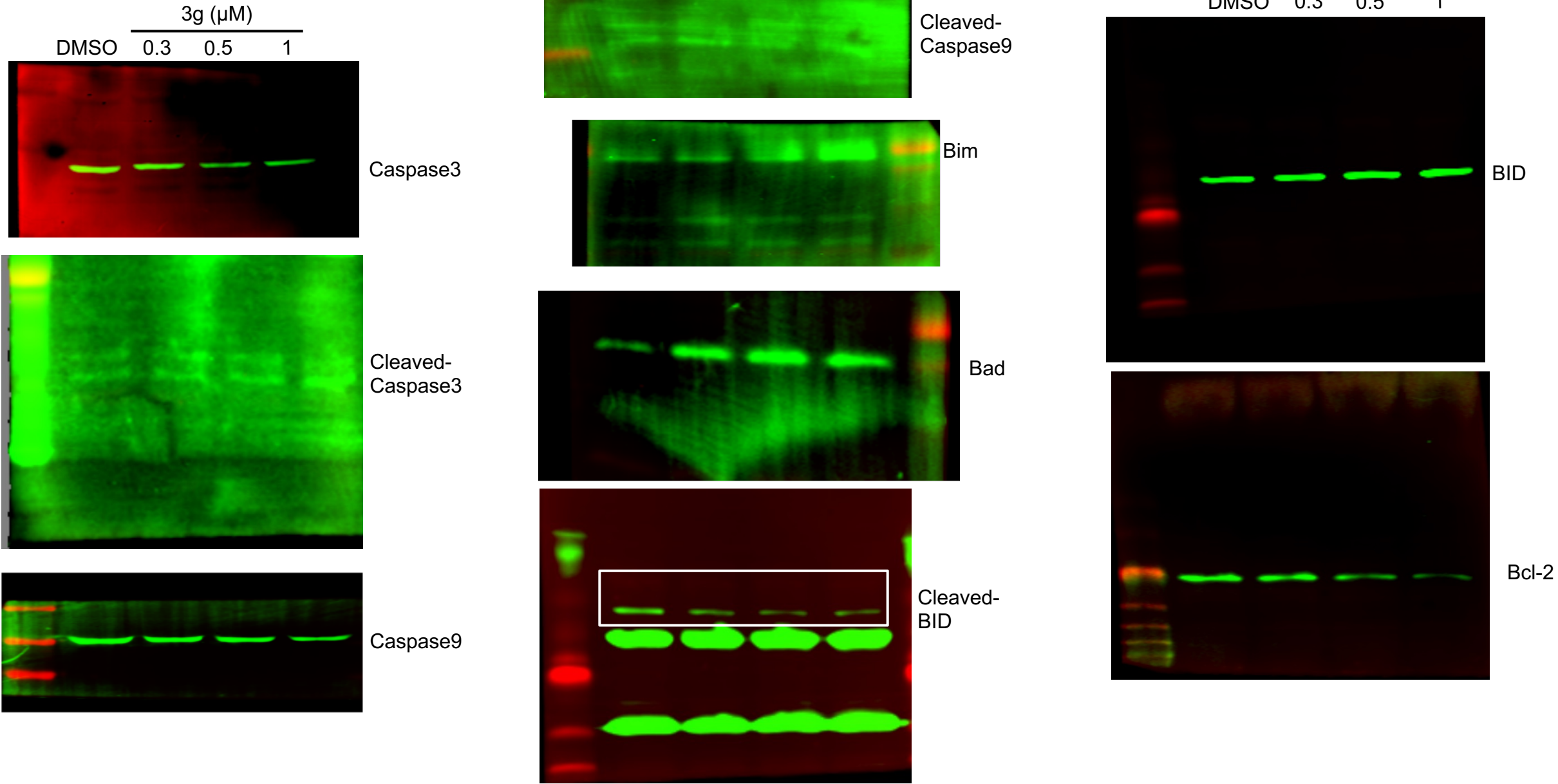

**Figure 4B**

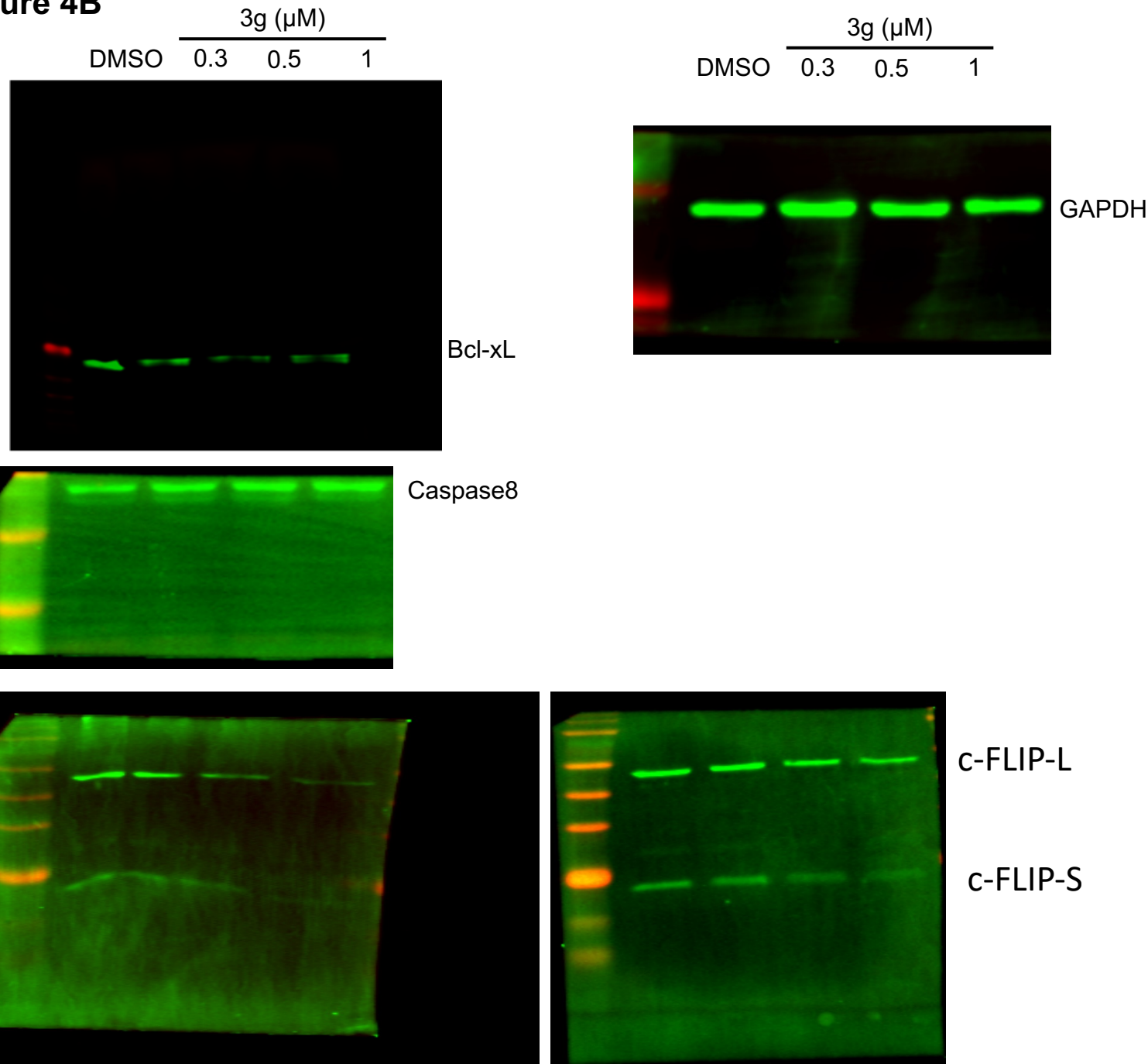

Figure 5B

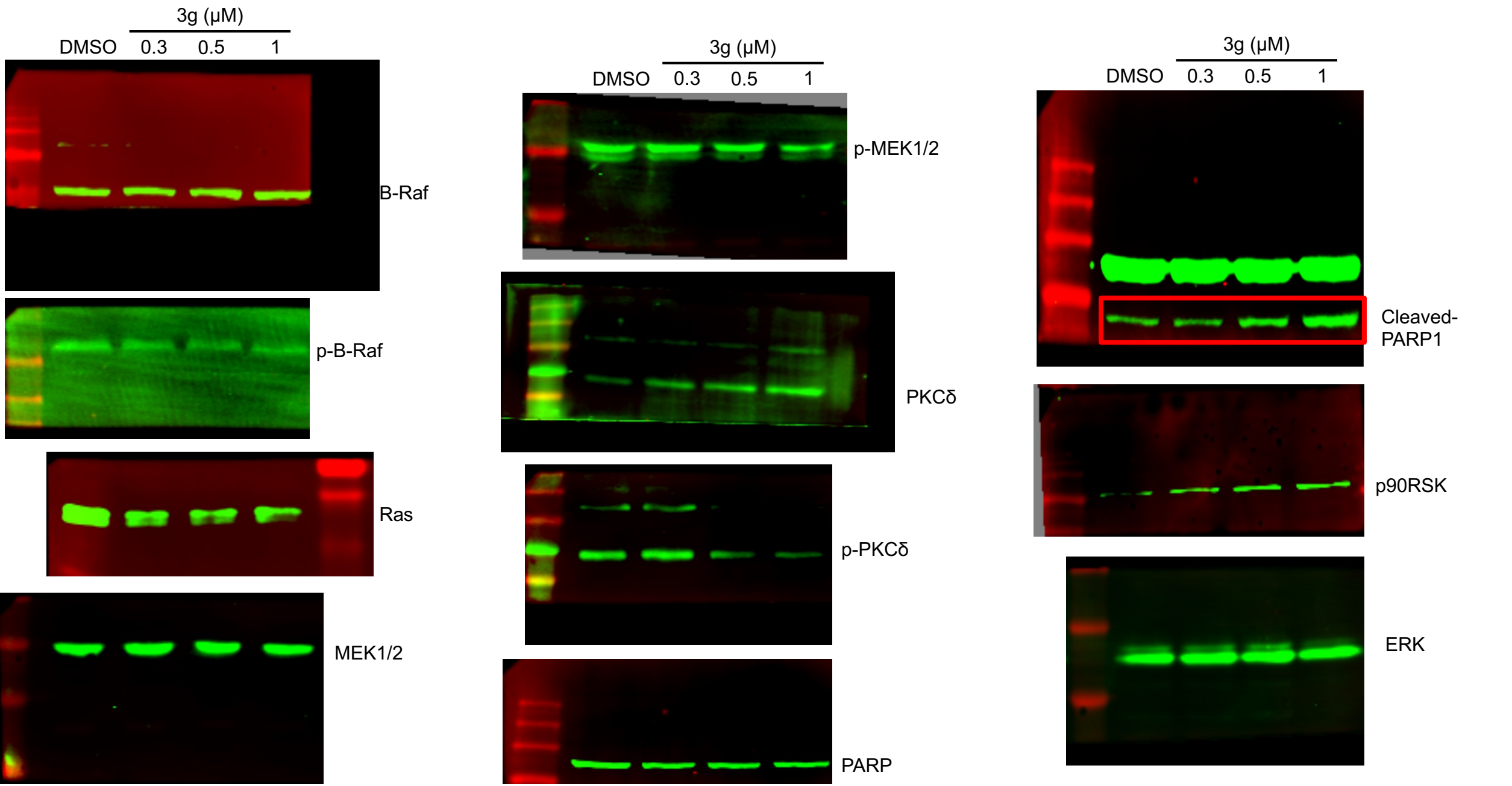

**Figure 5B**

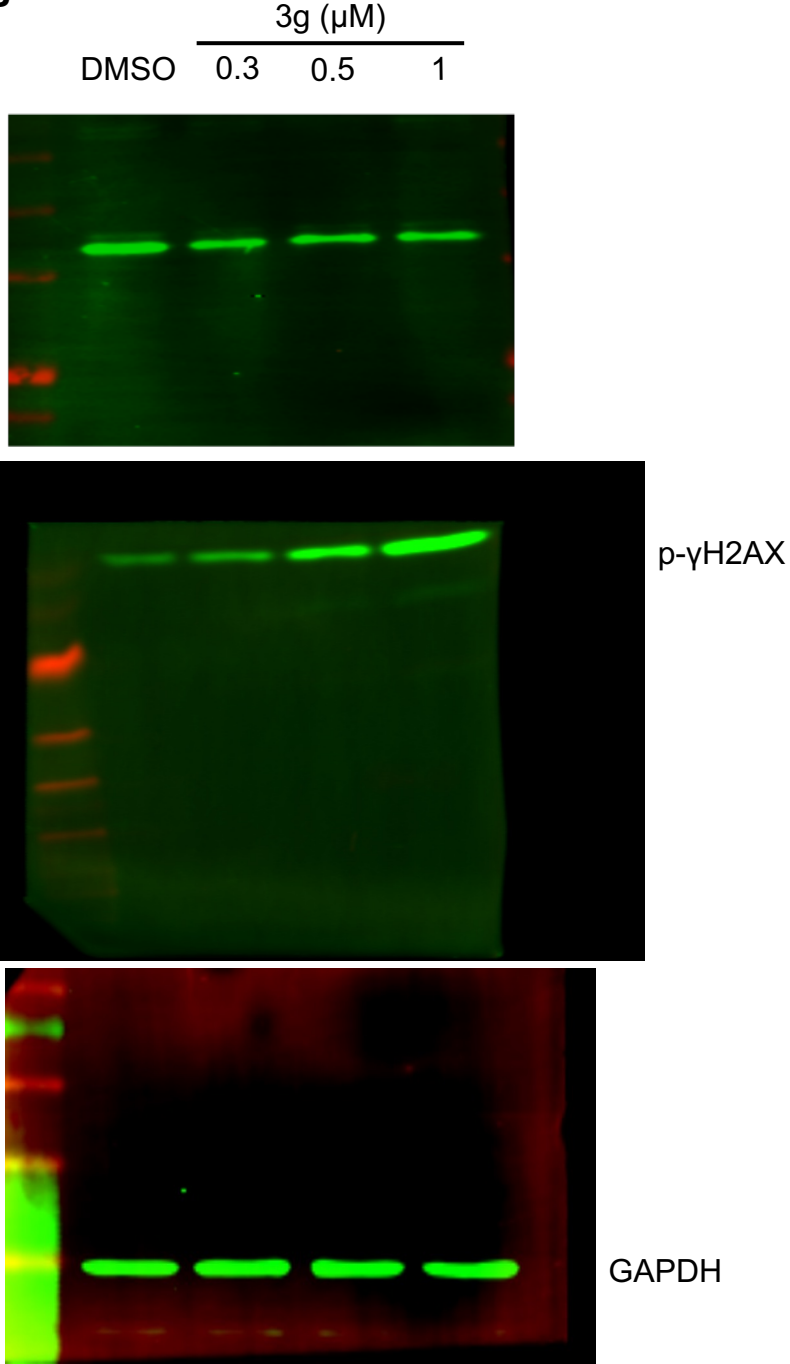

Supplement: Supplementary file 2 [file DataSheet4.PDF]
