# Supplementary material for: Design, Synthesis, and Antileukemic Evaluation of a Novel Mikanolide Derivative Through the Ras/Raf/MEK/ERK Pathway
Source: Front Pharmacol. 2022 May 20;13:809551. doi: 10.3389/fphar.2022.809551 (PMC9205396; doi:10.3389/fphar.2022.809551)
Supplement: Supplementary file 3 [file Presentation1.PPTX]

## Slide 1
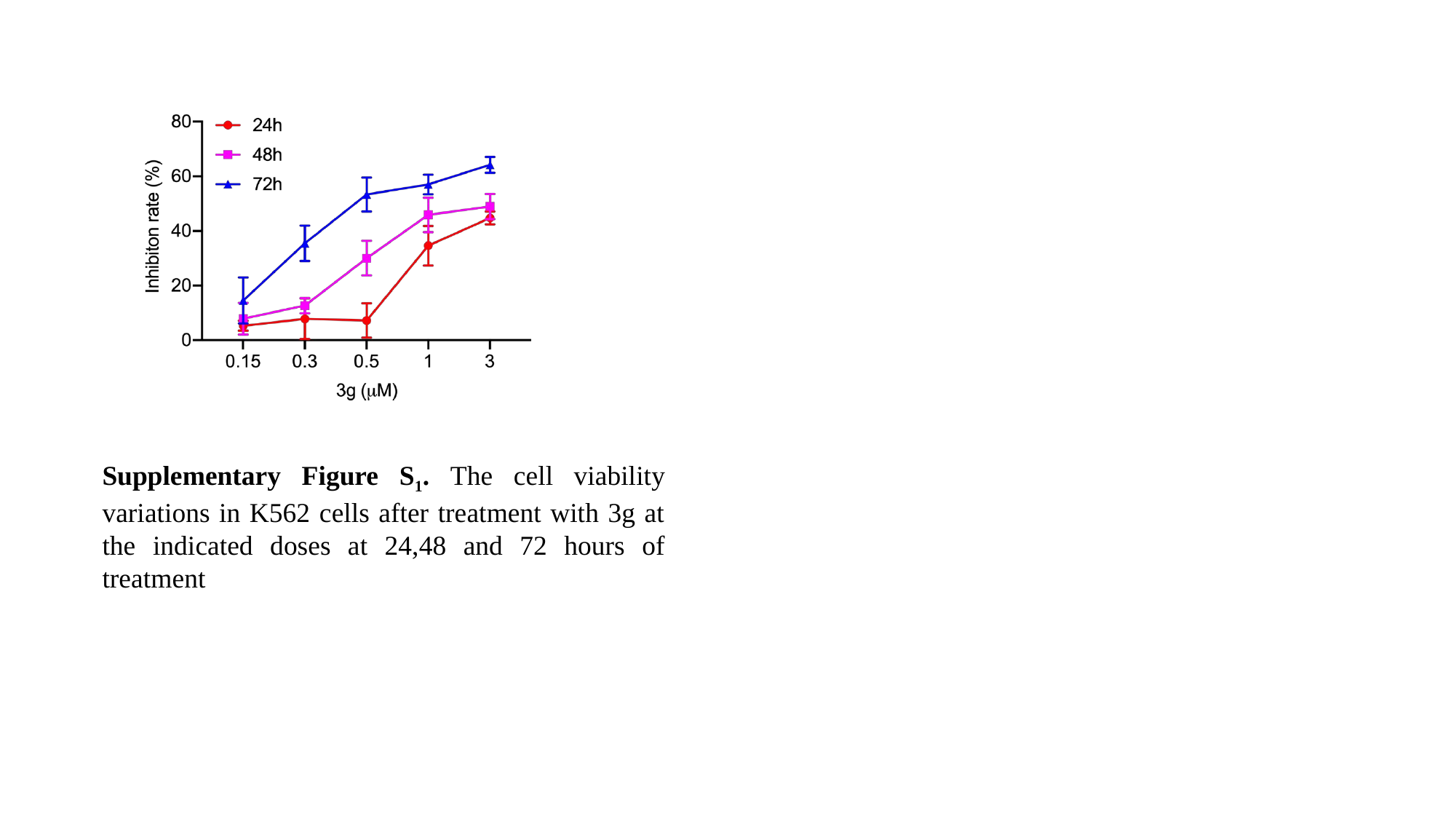

Supplementary Figure S1. The cell viability variations in K562 cells after treatment with 3g at the indicated doses at 24,48 and 72 hours of treatment

## Slide 2
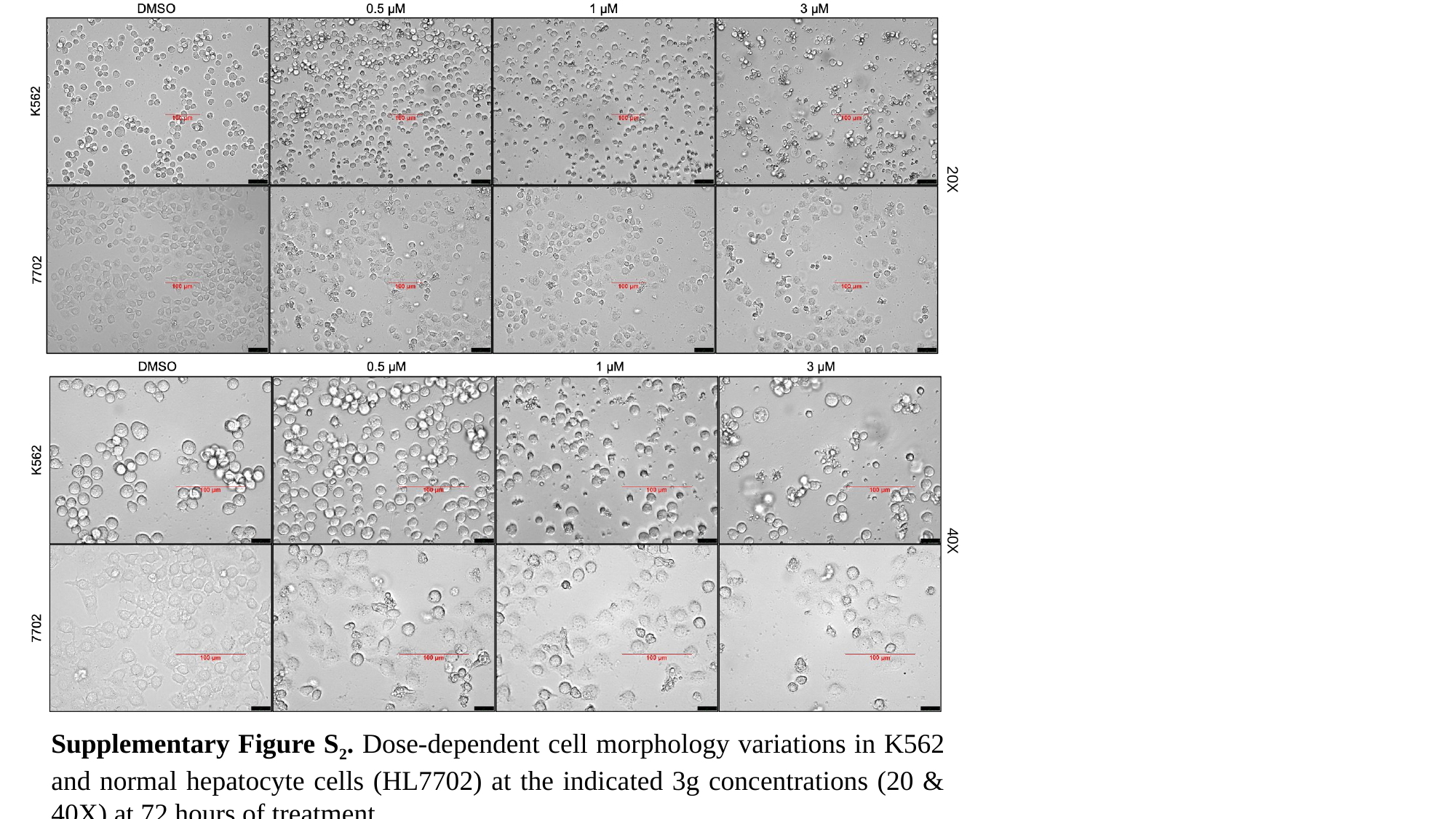

20X
40X
Supplementary Figure S2. Dose-dependent cell morphology variations in K562 and normal hepatocyte cells (HL7702) at the indicated 3g concentrations (20 & 40X) at 72 hours of treatment

## Slide 3
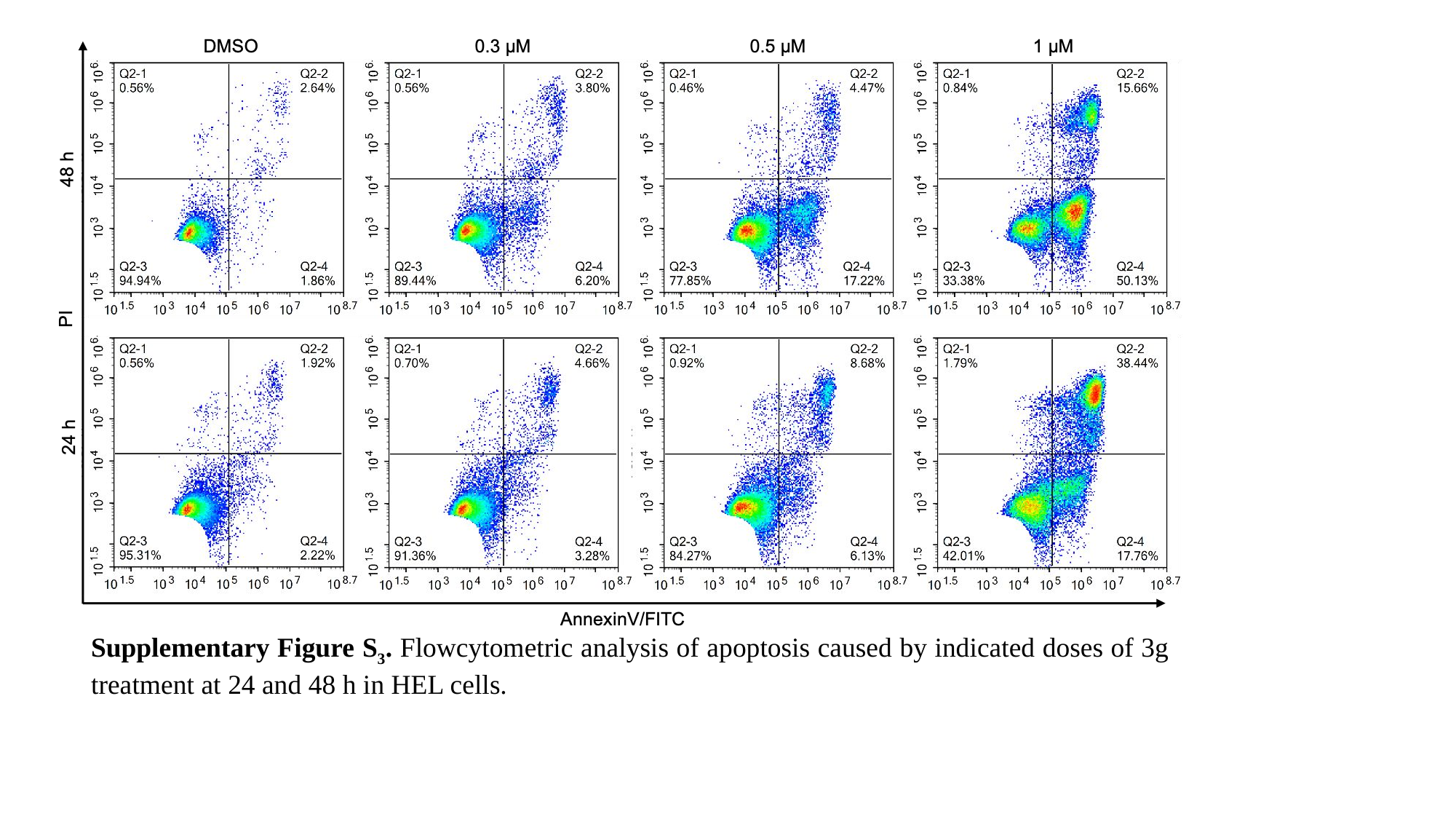

Supplementary Figure S3. Flowcytometric analysis of apoptosis caused by indicated doses of 3g treatment at 24 and 48 h in HEL cells.

## Slide 4
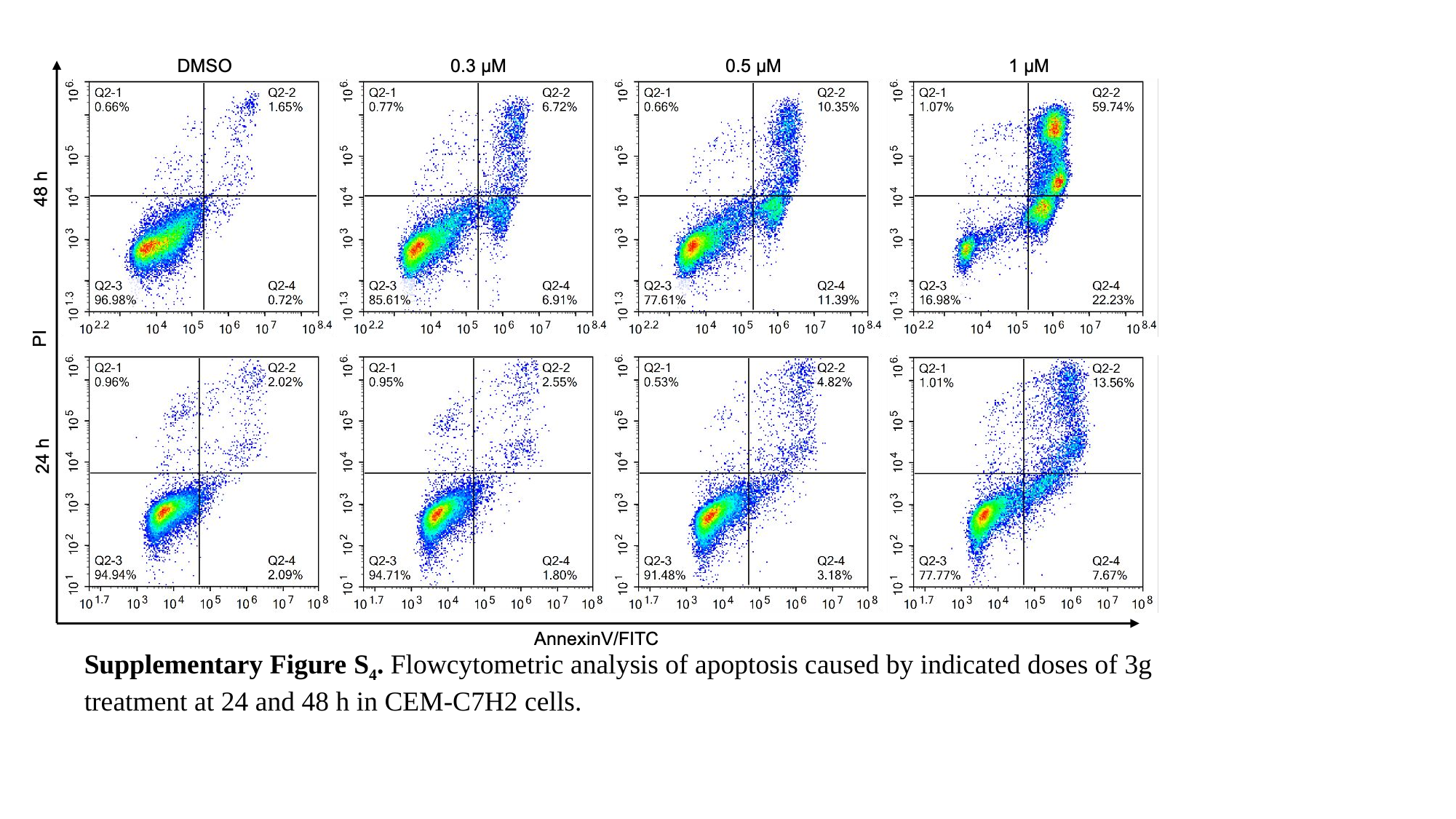

Supplementary Figure S4. Flowcytometric analysis of apoptosis caused by indicated doses of 3g treatment at 24 and 48 h in CEM-C7H2 cells.

## Slide 5
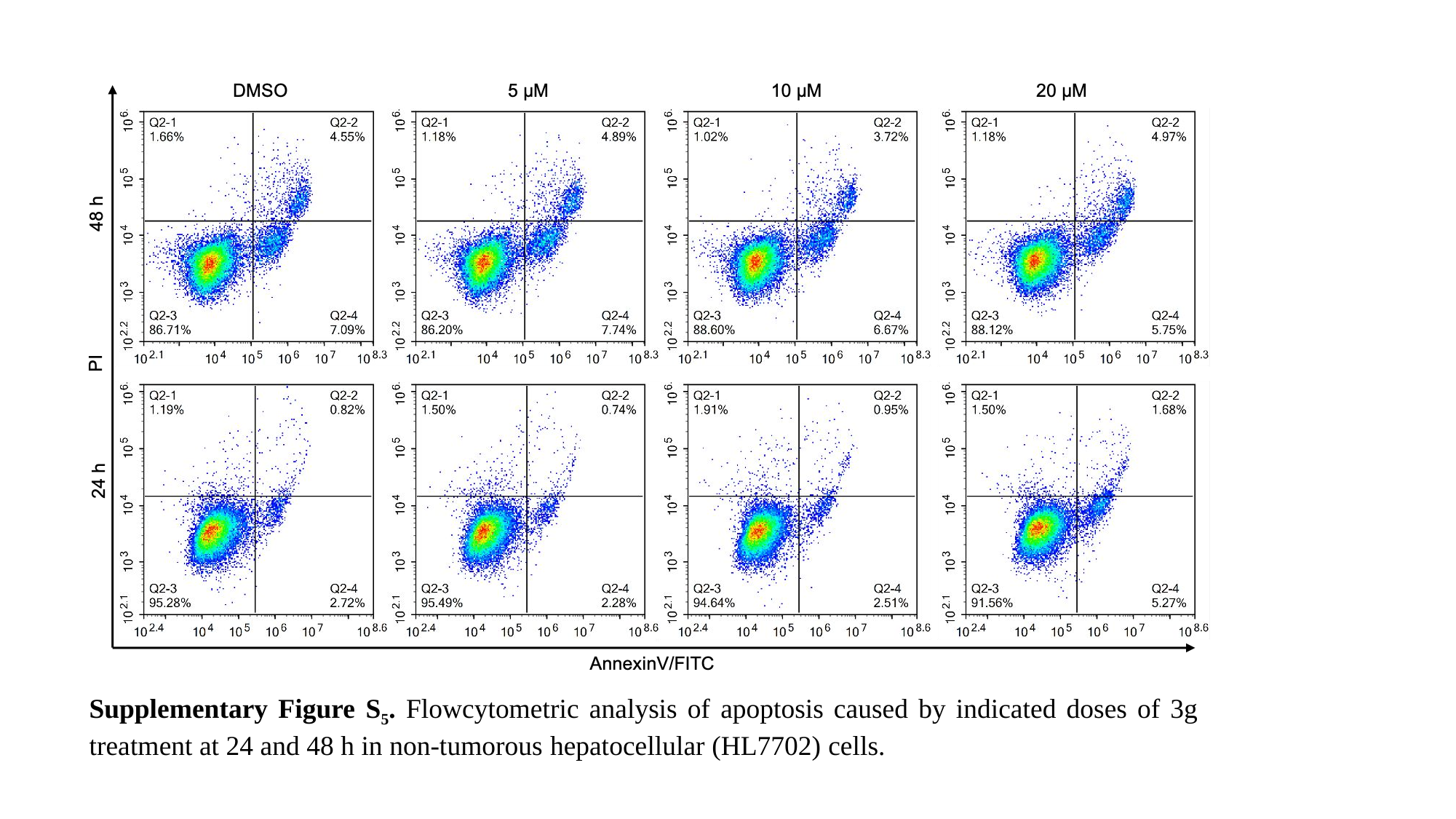

Supplementary Figure S5. Flowcytometric analysis of apoptosis caused by indicated doses of 3g treatment at 24 and 48 h in non-tumorous hepatocellular (HL7702) cells.

## Slide 6
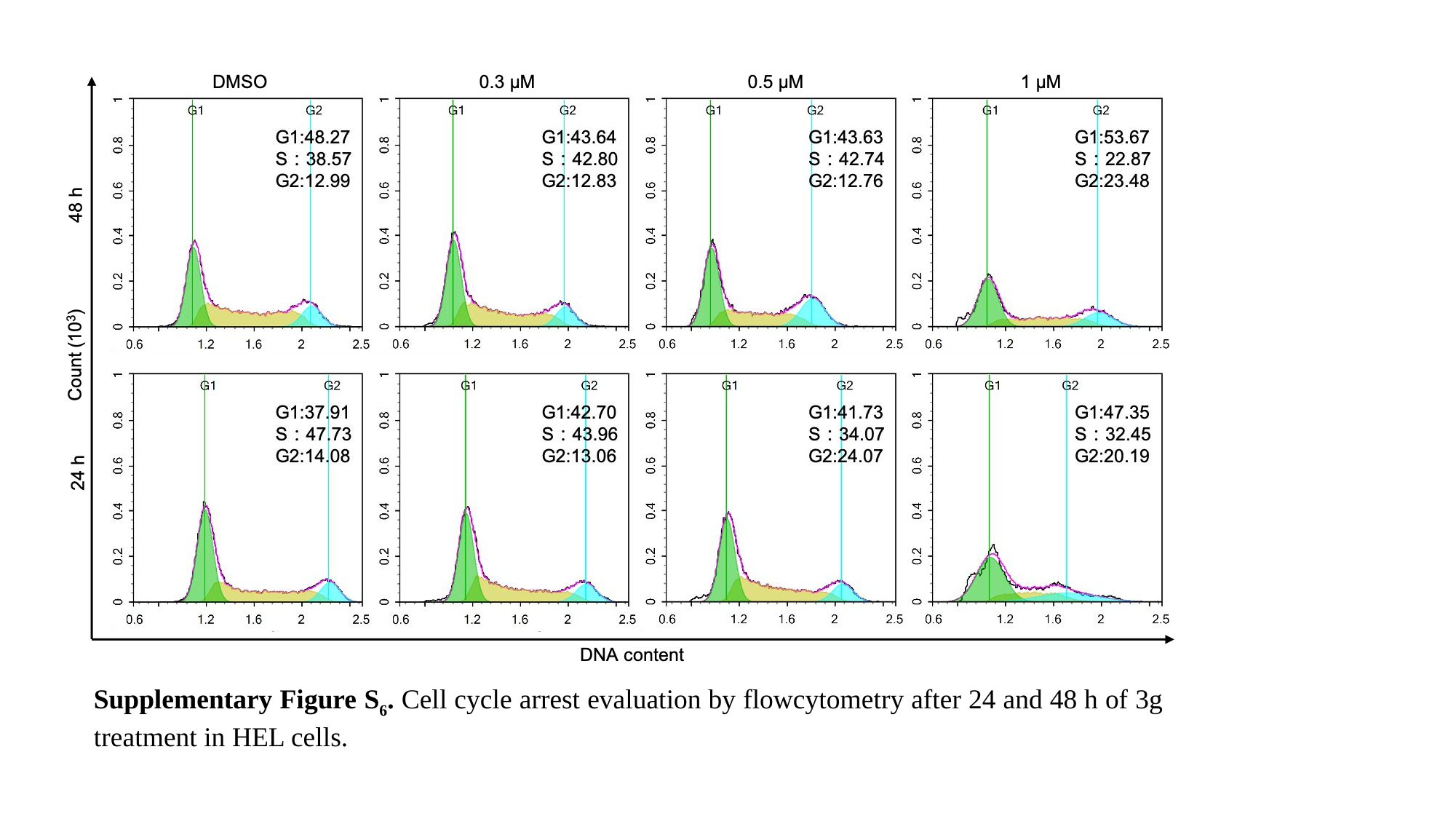

Supplementary Figure S6. Cell cycle arrest evaluation by flowcytometry after 24 and 48 h of 3g treatment in HEL cells.

## Slide 7
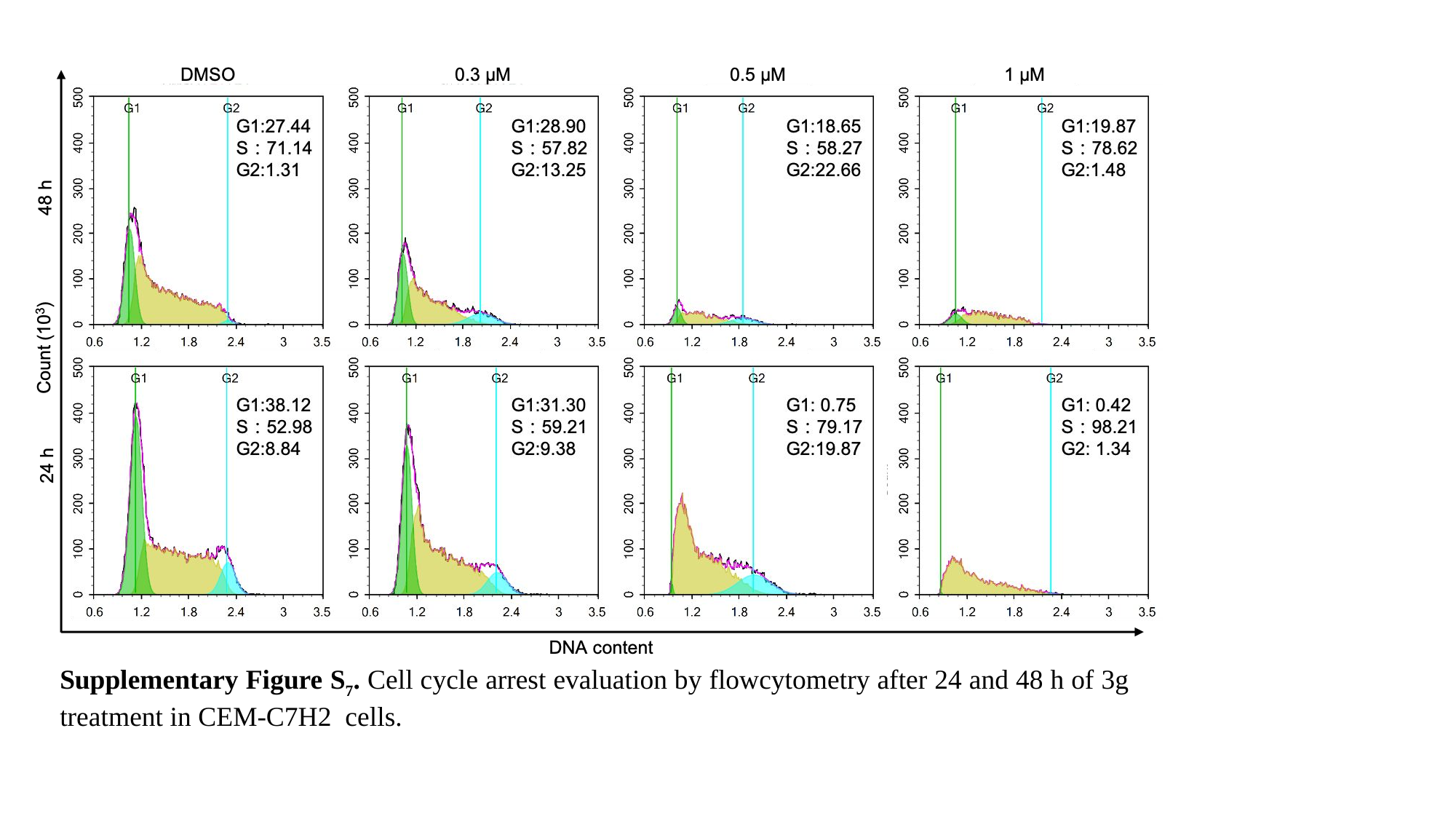

Supplementary Figure S7. Cell cycle arrest evaluation by flowcytometry after 24 and 48 h of 3g treatment in CEM-C7H2 cells.

## Slide 8
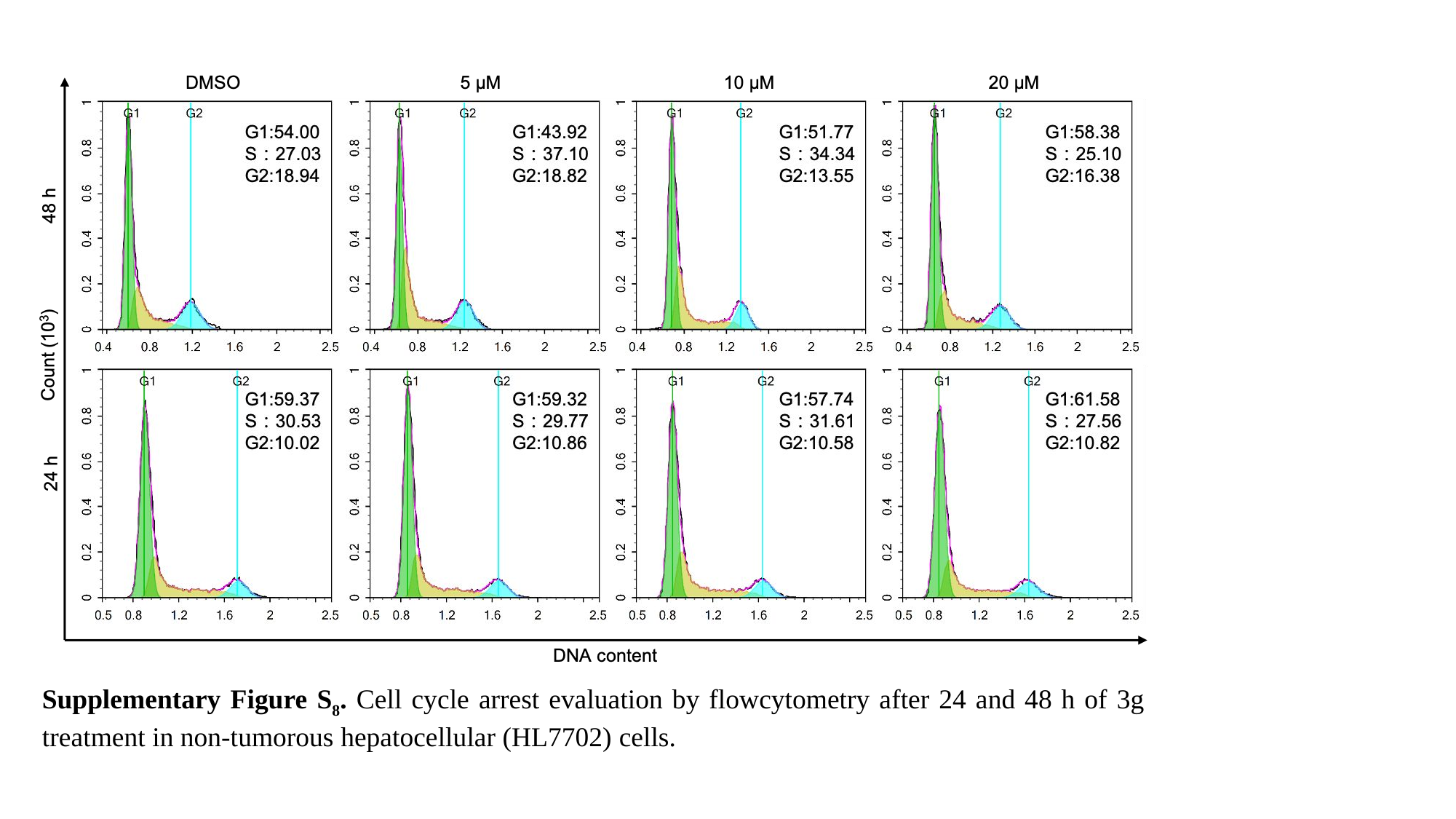

Supplementary Figure S8. Cell cycle arrest evaluation by flowcytometry after 24 and 48 h of 3g treatment in non-tumorous hepatocellular (HL7702) cells.
