# Supplementary material for: Design, Synthesis, and Antileukemic Evaluation of a Novel Mikanolide Derivative Through the Ras/Raf/MEK/ERK Pathway
Source: Front Pharmacol. 2022 May 20;13:809551. doi: 10.3389/fphar.2022.809551 (PMC9205396; doi:10.3389/fphar.2022.809551)
Supplement: Supplementary file 4 [file DataSheet5.DOCX]

Additional files are available at:

Supplementary Figures S_1_-S_8_: <https://www.jianguoyun.com/p/DVUdMcYQvqmDChjA3rAE>

Supplementary material: <https://www.jianguoyun.com/p/DR25GFcQvqmDChiMiJsE>

Apoptotic analysis (.fcs): <https://www.jianguoyun.com/p/DYwdhtsQvqmDChjAiJsE>

Cell cycle analysis (.fcs): <https://www.jianguoyun.com/p/DZLRDFwQvqmDChi7iJsE>

Mitochondrial membrane potential: <https://www.jianguoyun.com/p/Db99wD4QvqmDChi2iJsE>

Hoechst Staining: <https://www.jianguoyun.com/p/DblpwsQQvqmDChi5iJsE>

Data sheet: <https://www.jianguoyun.com/p/DV5hsGsQvqmDChiyiJsE>

Flow cytometry gating strategy: <https://www.jianguoyun.com/p/Dd_u47cQvqmDChi0iJsE>

Uncropped Western blot images: <https://www.jianguoyun.com/p/DeJv-3oQvqmDChiziJsE>

3g & positive control treated cells images: <https://www.jianguoyun.com/p/DdSU5vsQvqmDChjY2rAE>
