# Supplementary material for: Design, Synthesis, and Antileukemic Evaluation of a Novel Mikanolide Derivative Through the Ras/Raf/MEK/ERK Pathway
Source: Front Pharmacol. 2022 May 20;13:809551. doi: 10.3389/fphar.2022.809551 (PMC9205396; doi:10.3389/fphar.2022.809551)
Supplement: Supplementary file 6 [file DataSheet1.DOCX]

***Supplementary Material***

Nuclear magnetic and high resolution mass spectra of synthesized compounds
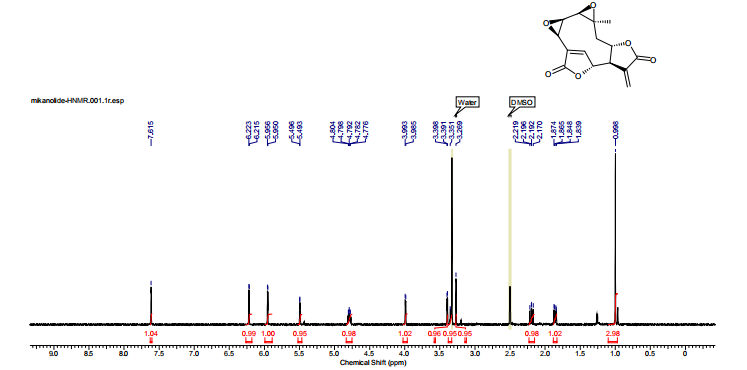


**1**


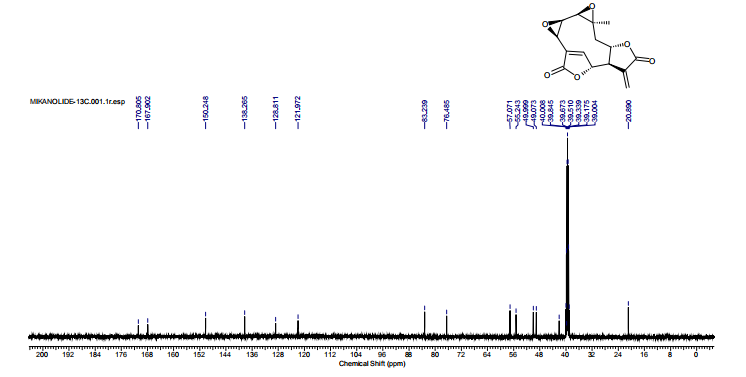


**1**


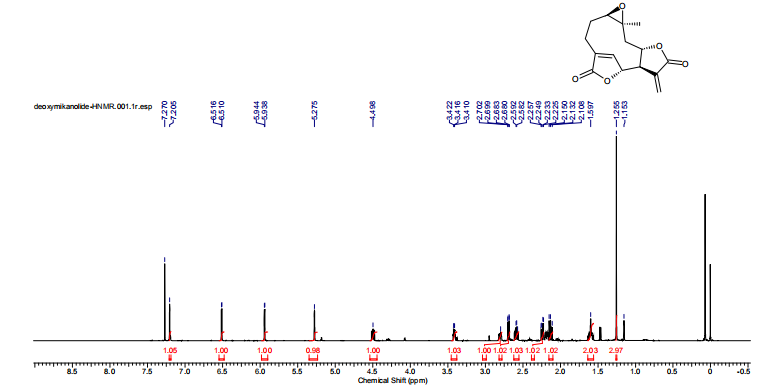


**2**


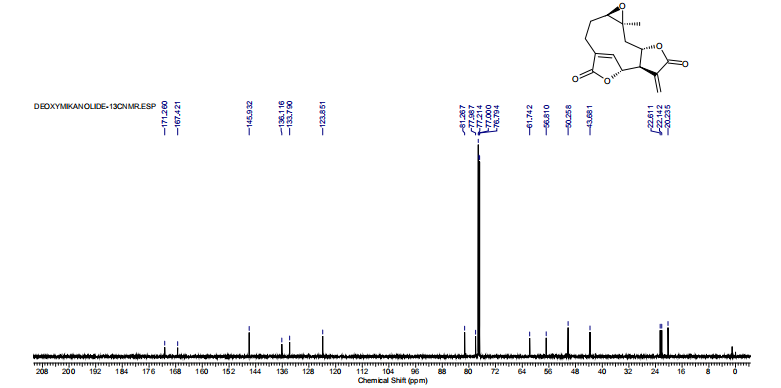

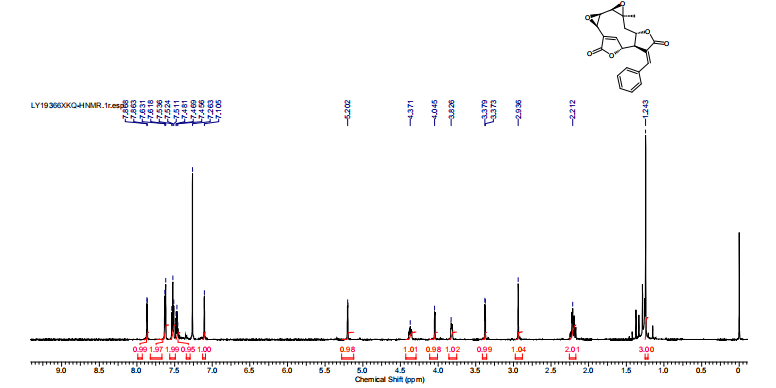

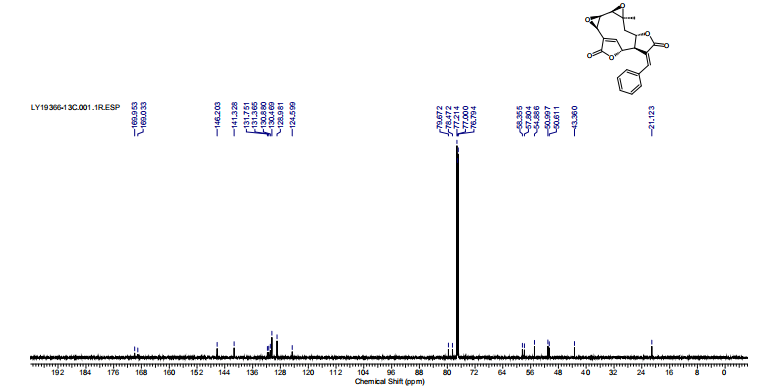


**3a**

**2**

**3a**

**3a**


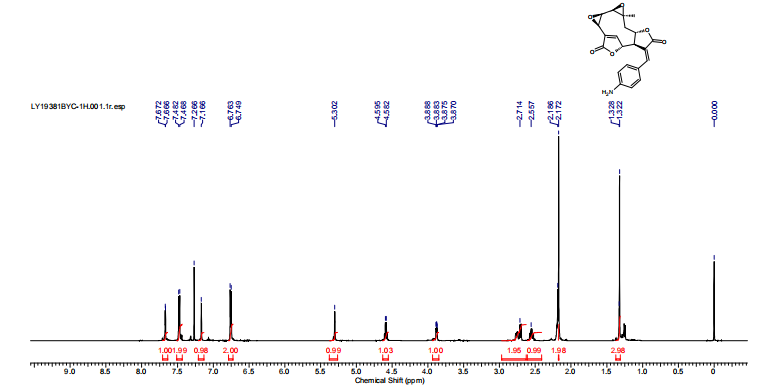

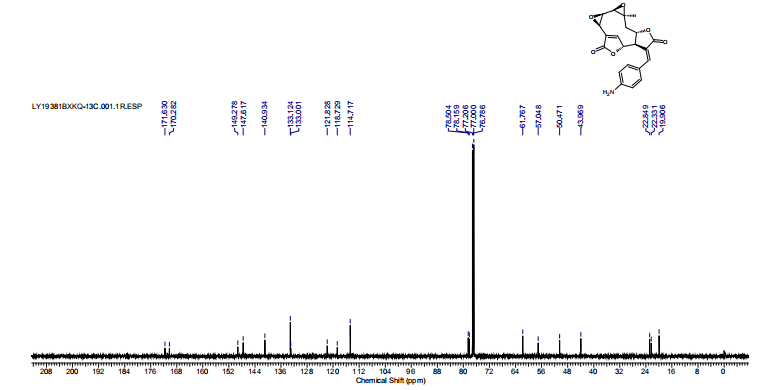


**3b**

**3b**


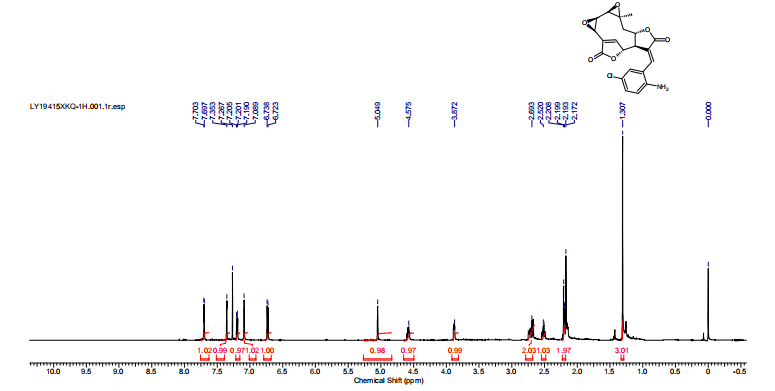

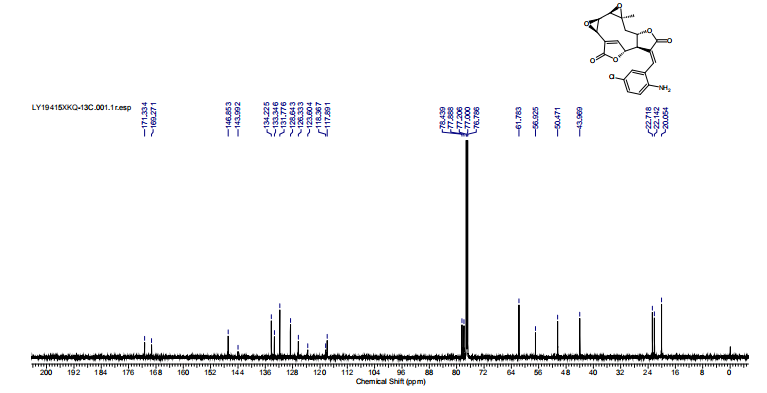

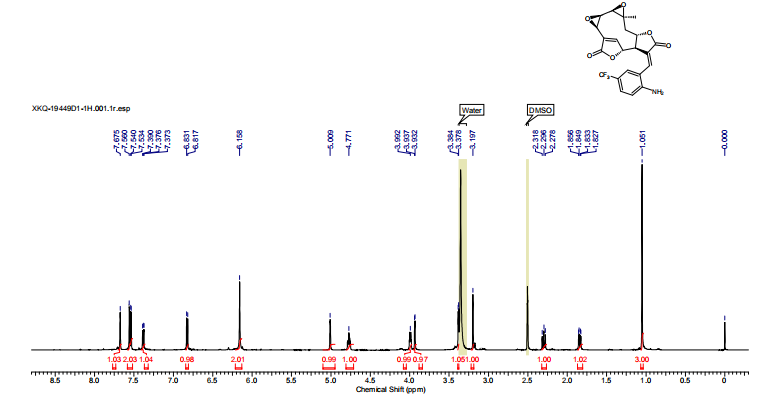

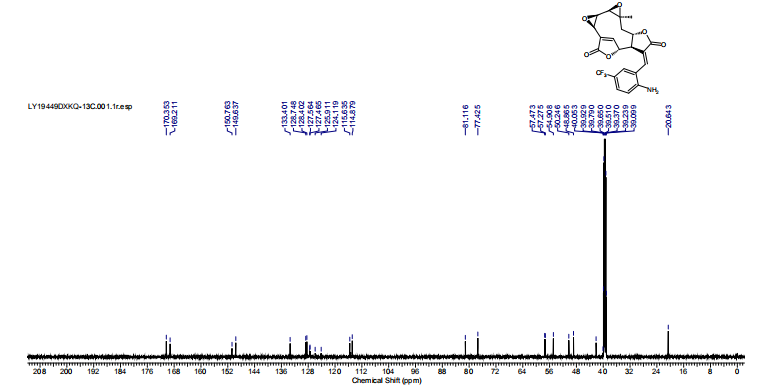

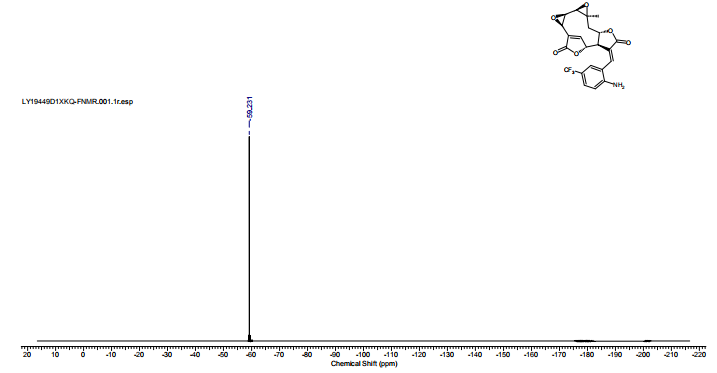

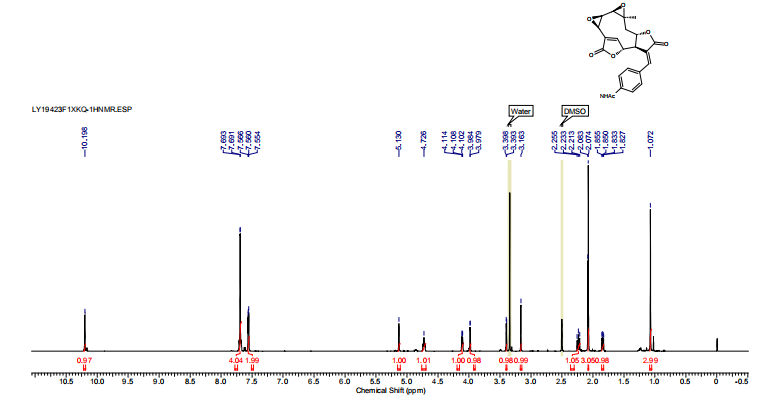

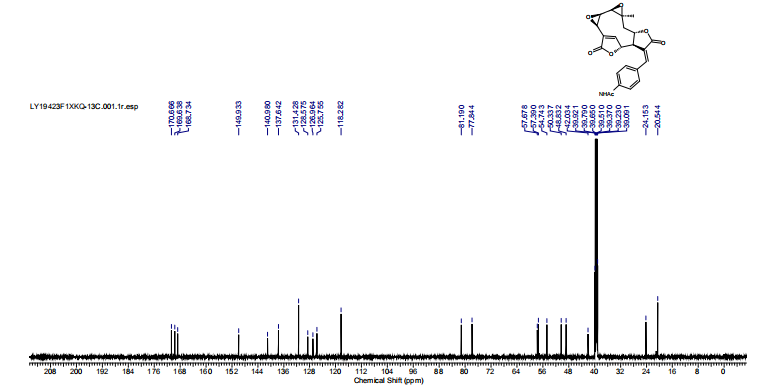


**3c**

**3c**

**3b**

**3c**

**3d**

**3d**

**3d**

**3d**

**3e**

**3e**

**3e**


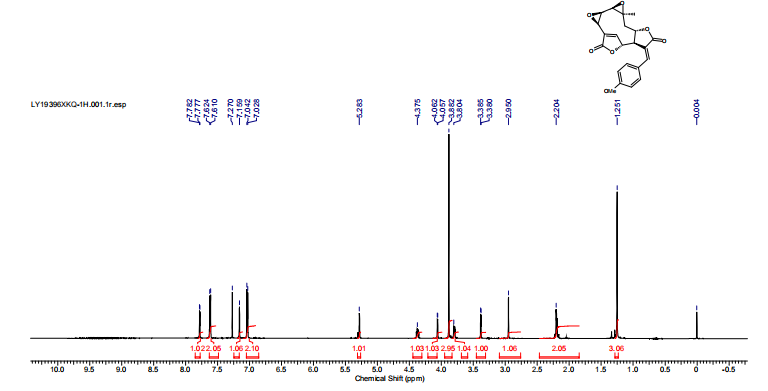

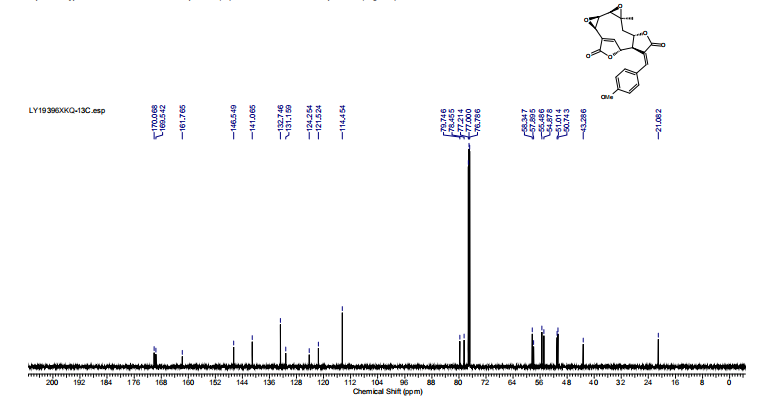


**3f**

**3f**

**3f**


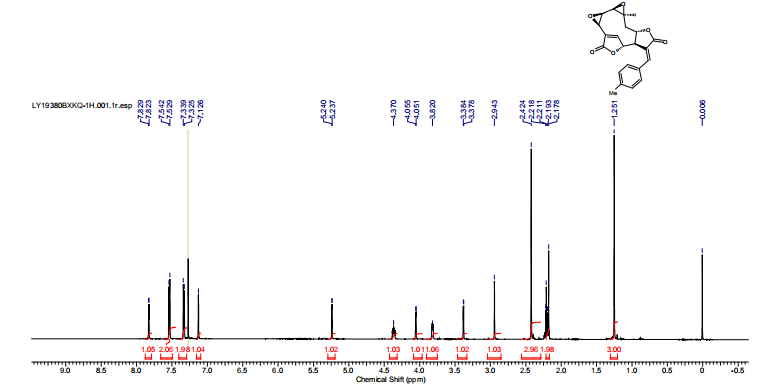

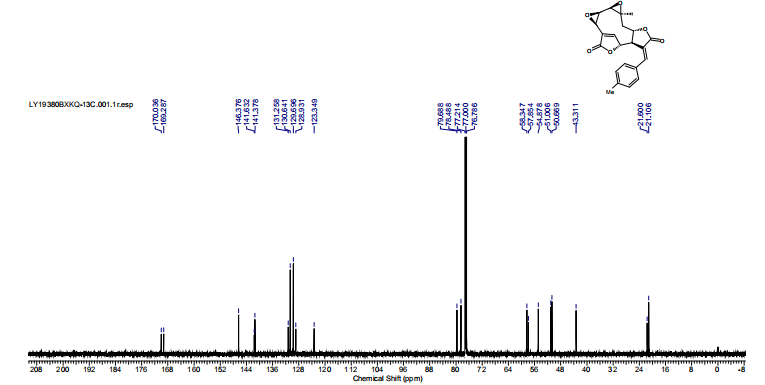

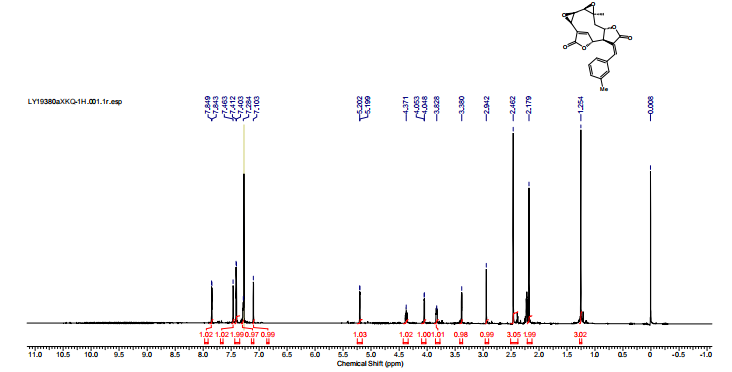

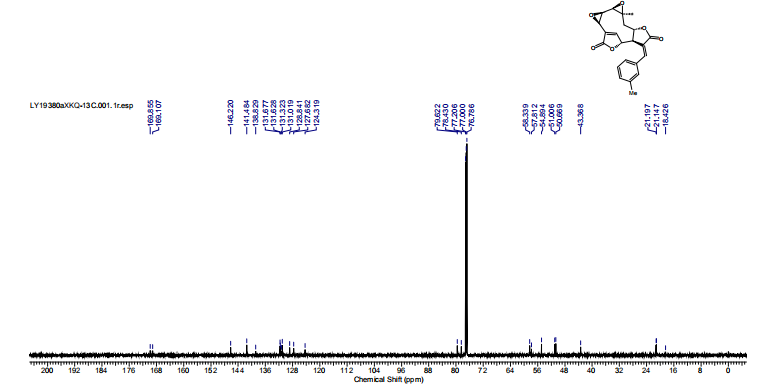

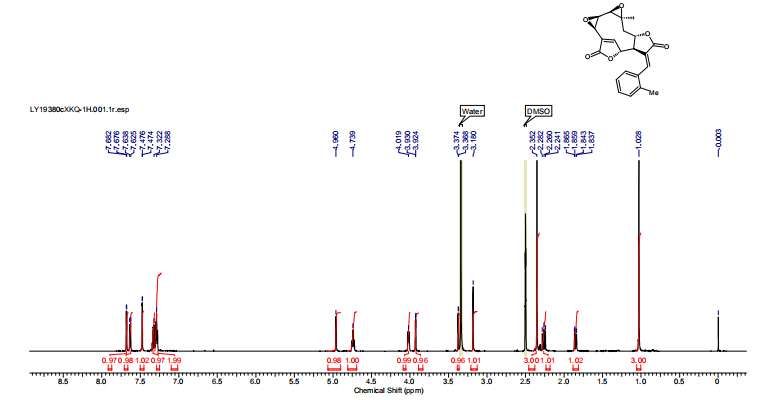

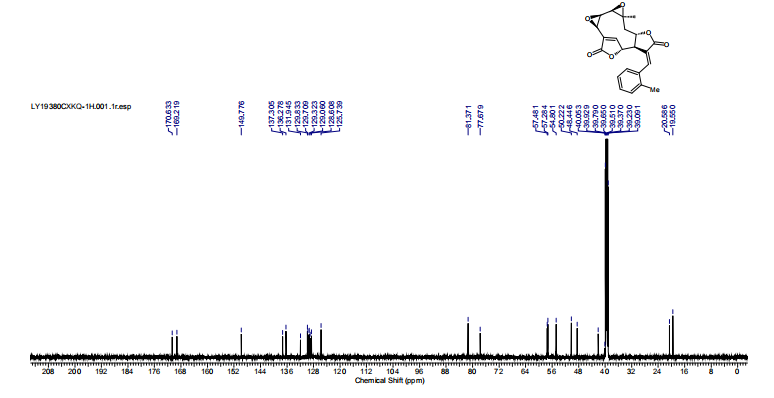

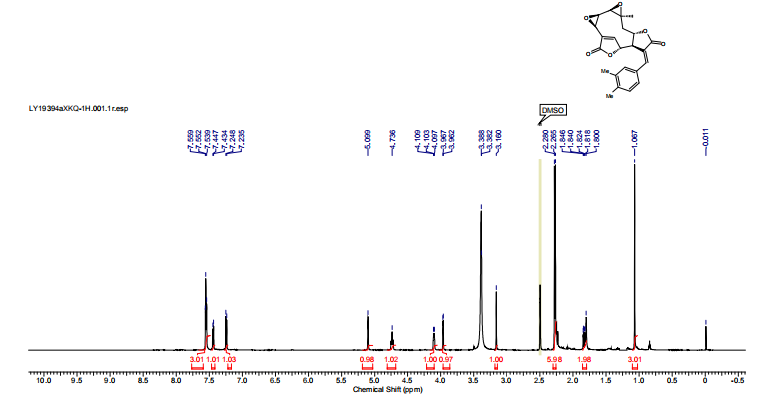

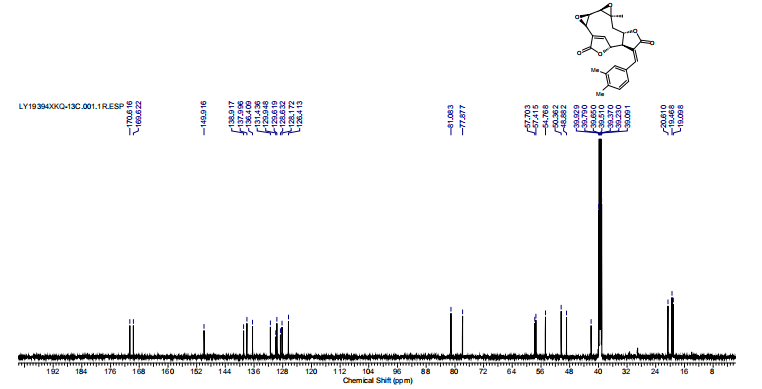


**3g**

**3g**

**3g**

**3h**

**3h**

**3h**

**3i**

**3i**

**3i**

**3j**

**3j**

**3j**


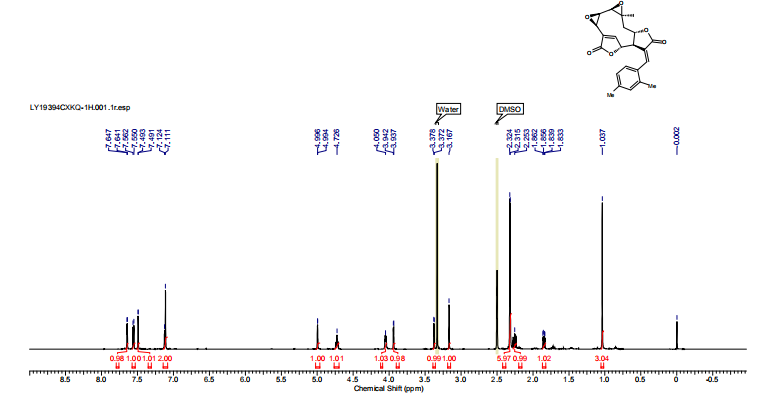

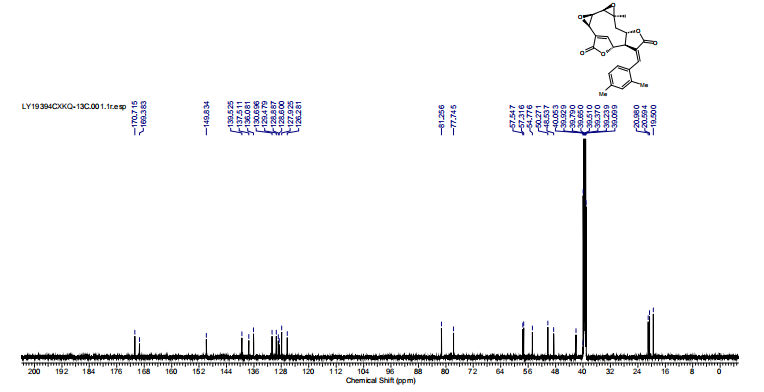


**3k**

**3k**

**3k**


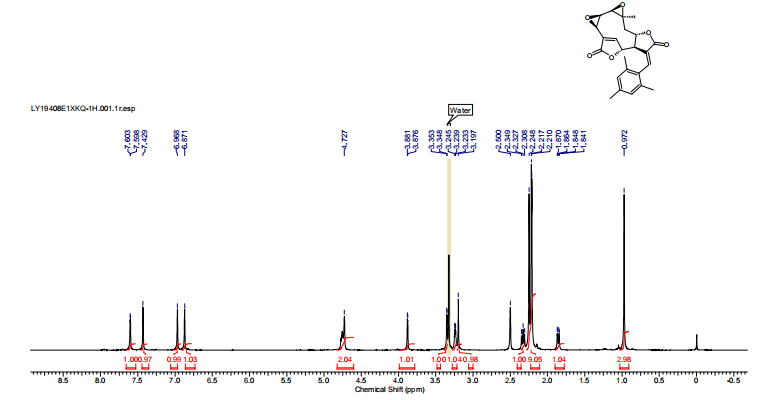

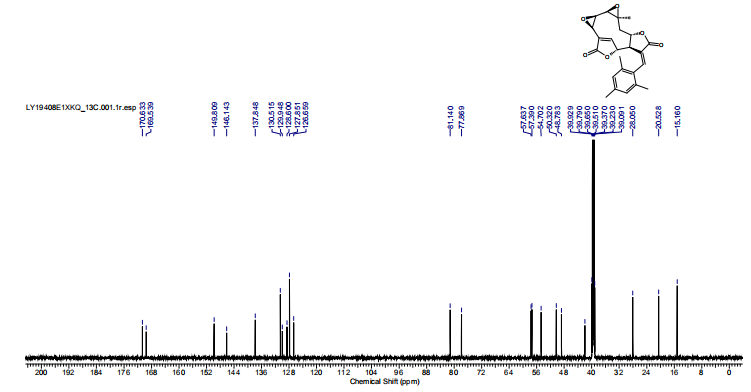


**3l**

**3l**

**3l**


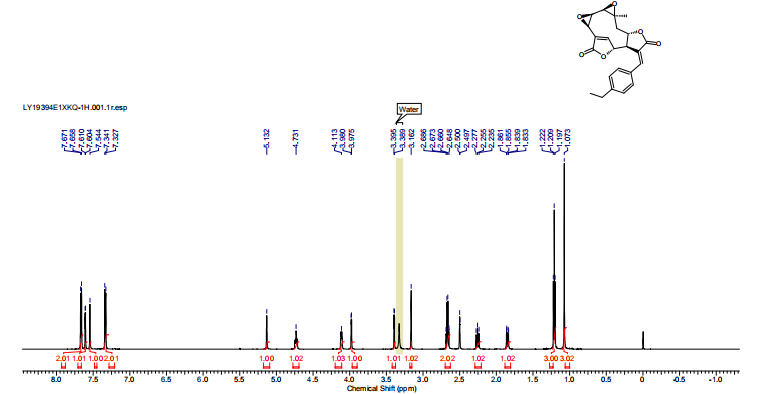

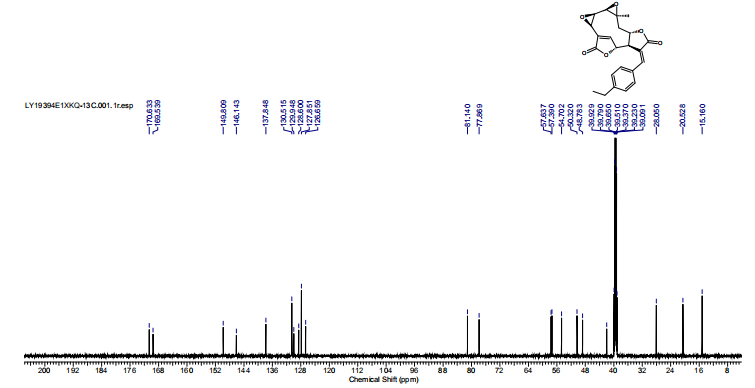


**3m**

**3m**

**3m**


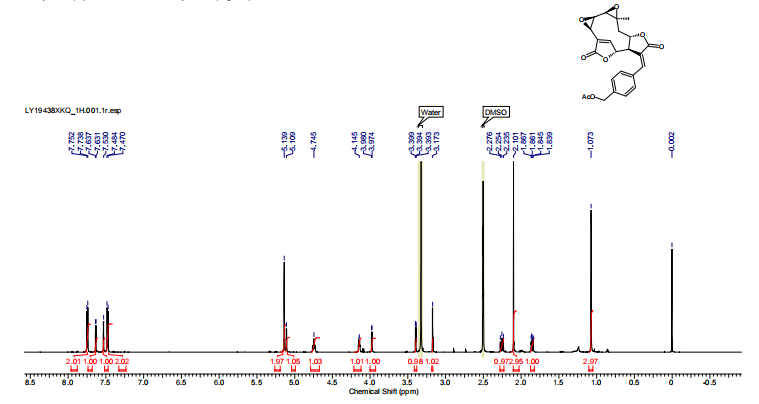

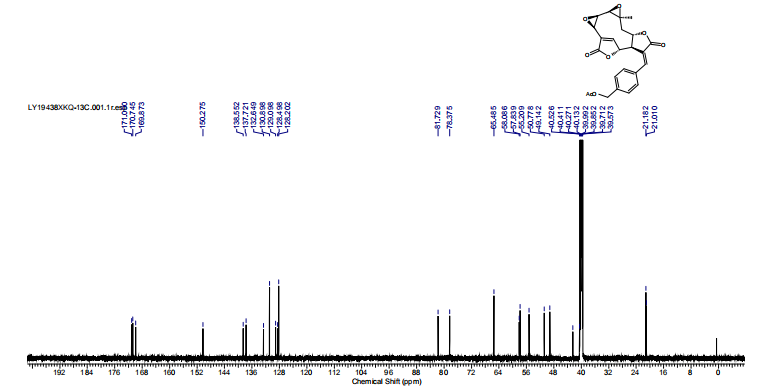


**3n**

**3n**

**3n**


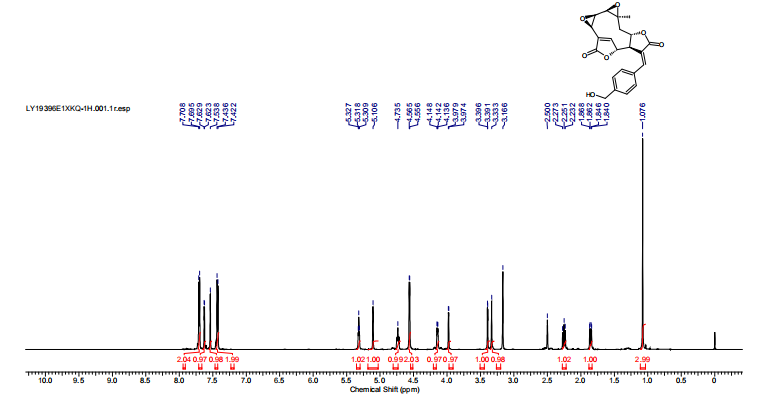

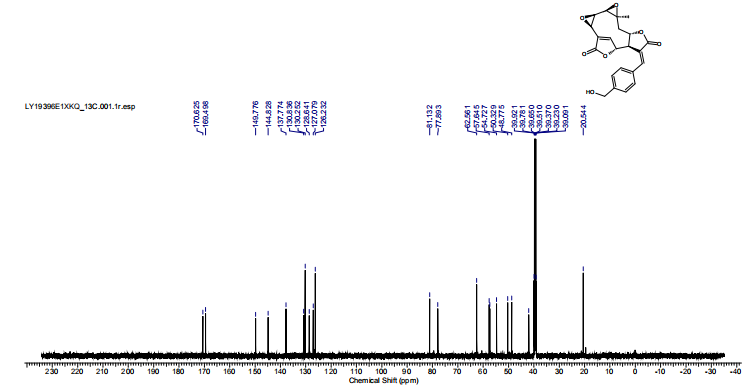

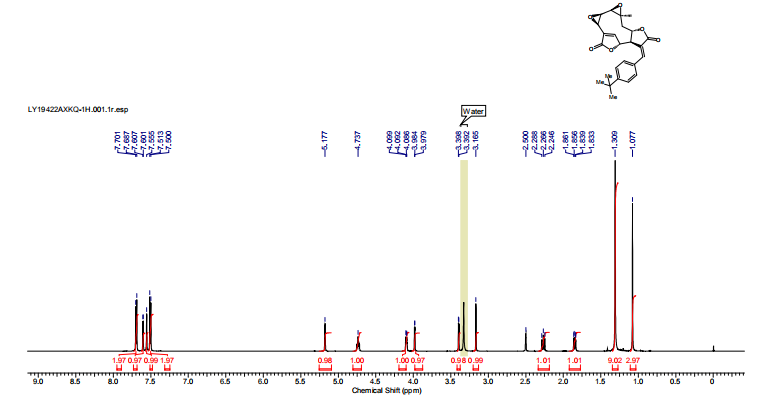

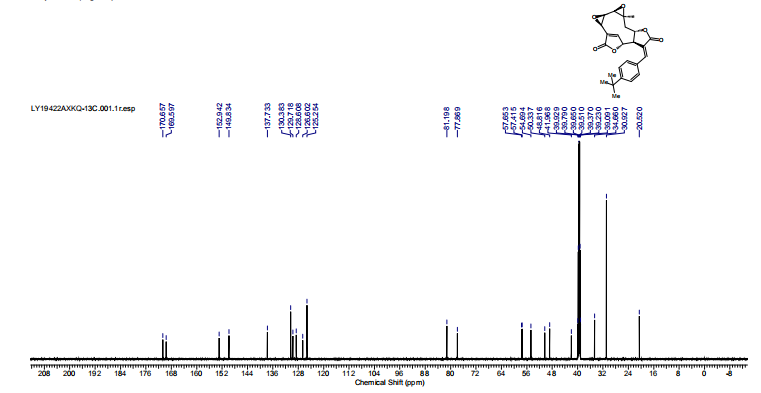


**3o**

**3o**

**3o**

**3p**

**3p**

**3p**


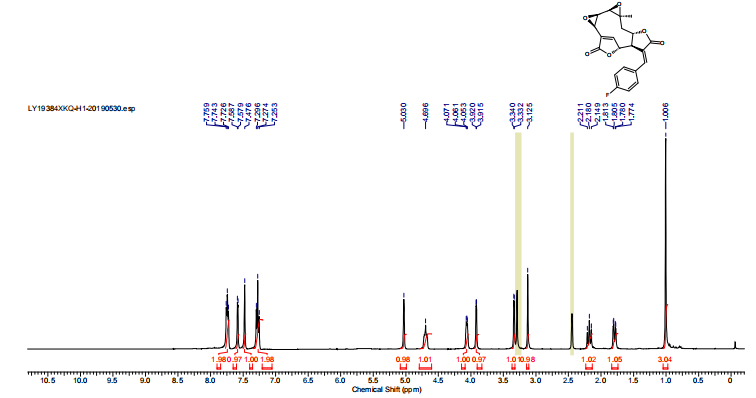

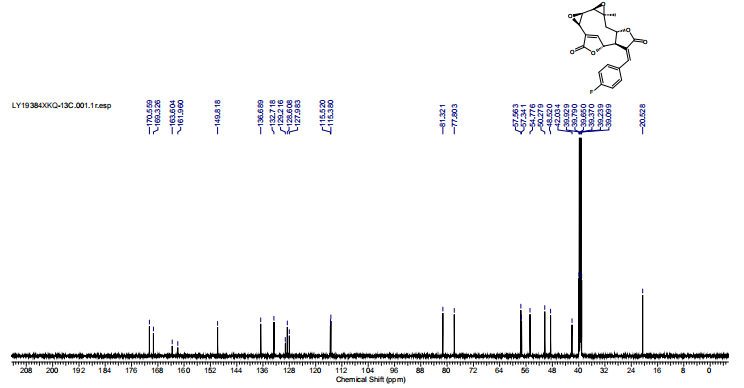

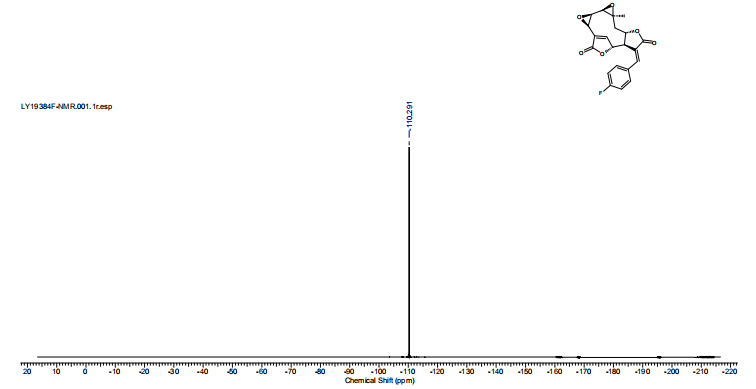

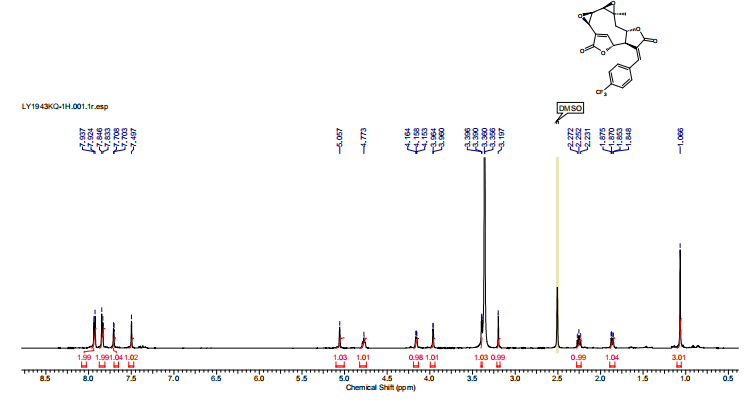

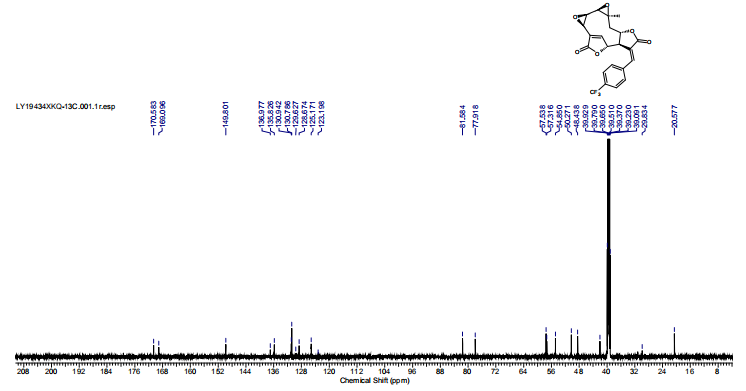

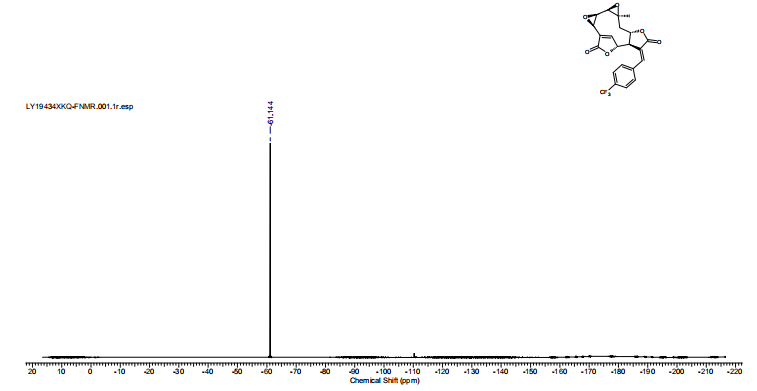

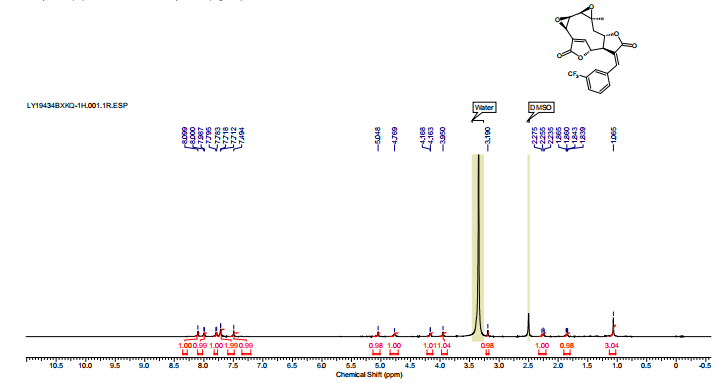

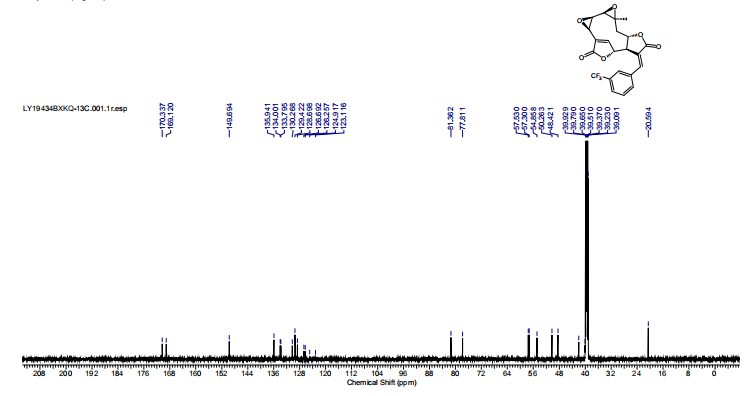

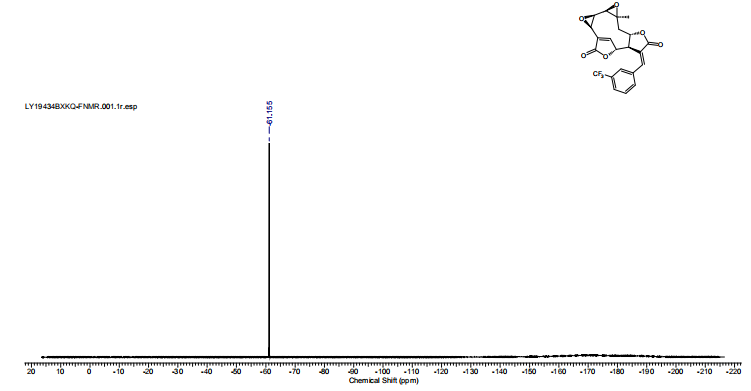

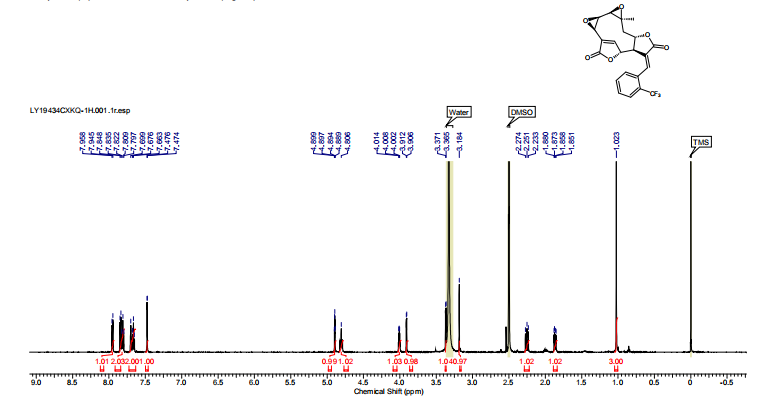

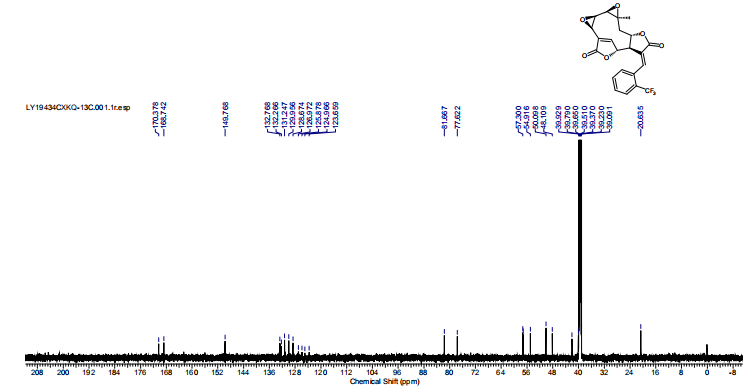

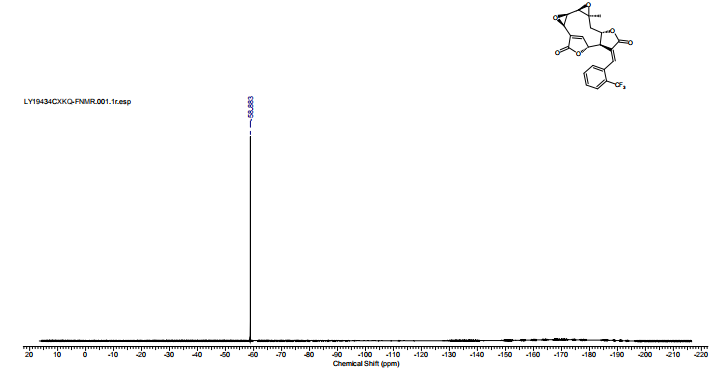

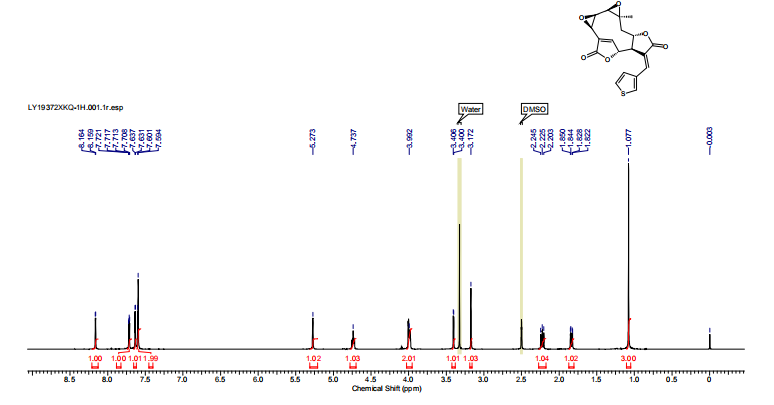

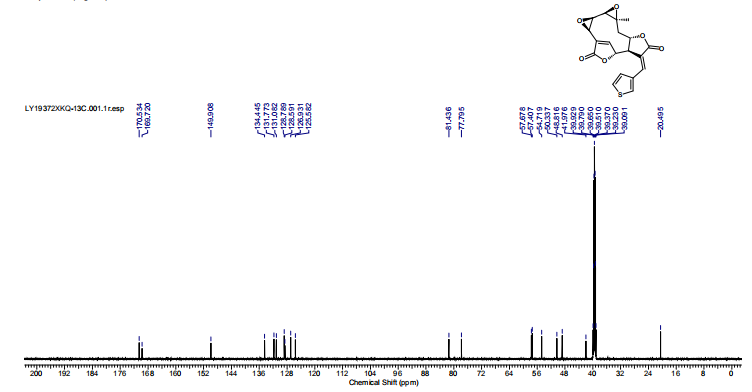

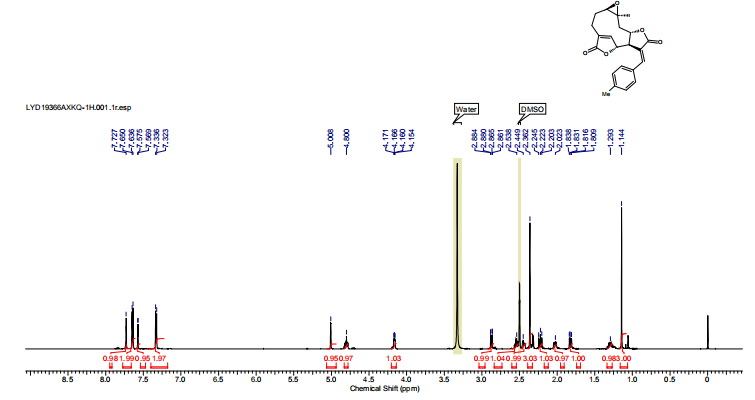

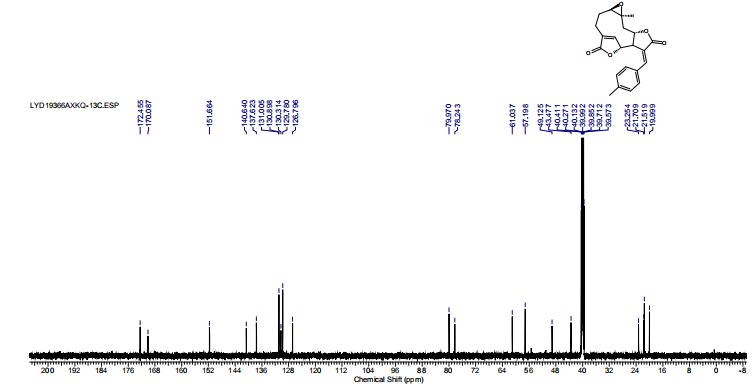


**3q**

**3q**

**3q**

**3q**

**3r**

**3r**

**3r**

**3r**

**3s**

**3s**

**3s**

**3s**

**3t**

**3t**

**3t**

**3t**

**3u**

**3u**

**3u**

**4**

**4**

**4**
